# Supplementary figures and images for: Reduced soil fauna decomposition in a high background radiation area
Source: PLoS One. 2021 Mar 17;16(3):e0247793. doi: 10.1371/journal.pone.0247793 (PMC7968631; doi:10.1371/journal.pone.0247793)

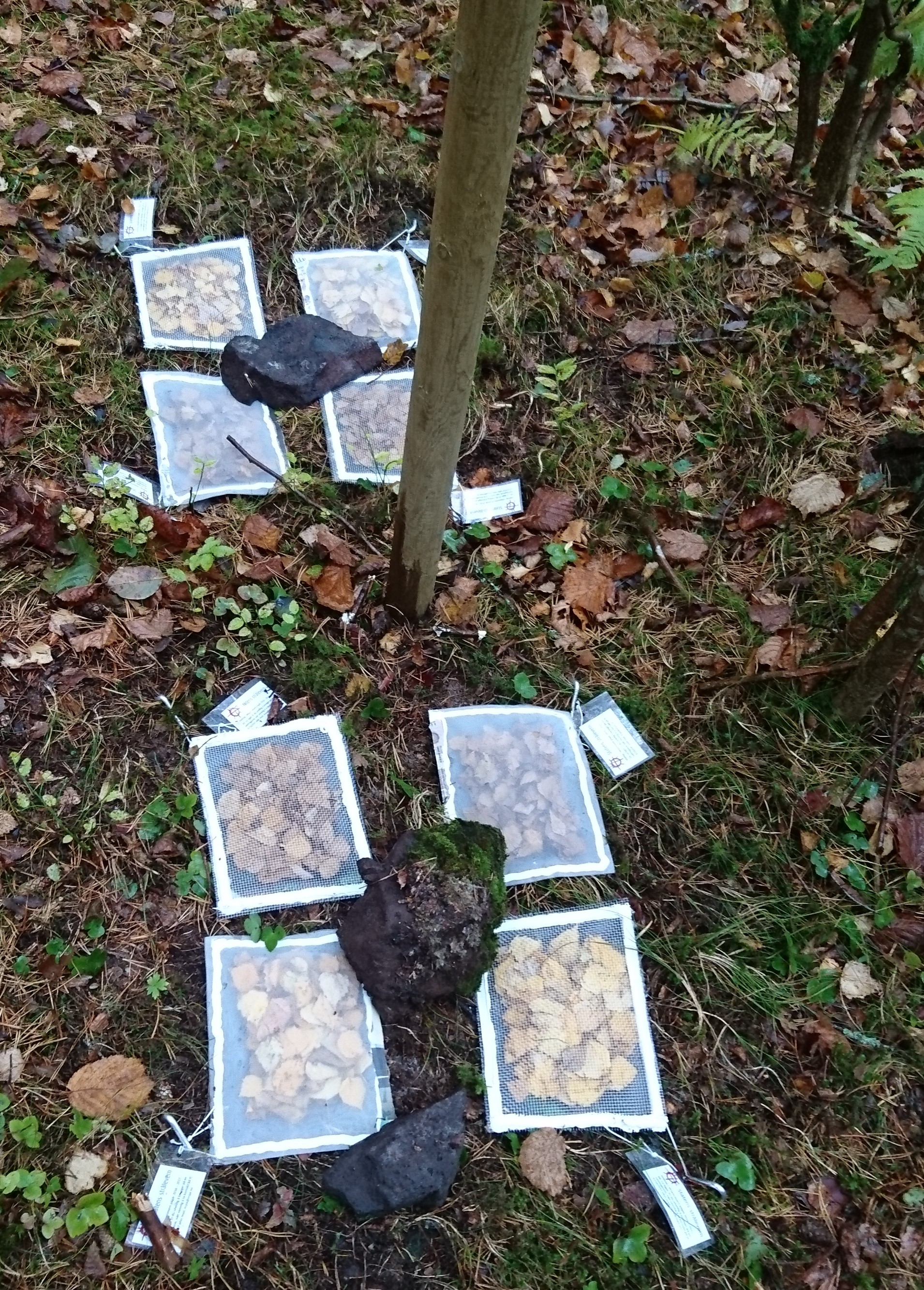

Supplement: S1 Graphical abstract — (JPG) [file pone.0247793.s001.jpg]
