# Supplementary material for: Reduced soil fauna decomposition in a high background radiation area
Source: PLoS One. 2021 Mar 17;16(3):e0247793. doi: 10.1371/journal.pone.0247793 (PMC7968631; doi:10.1371/journal.pone.0247793)
Supplement: Attachment — Submitted filename: PONE-D-20-38999_with_reviewer comments_02Jan2021.pdf [file pone.0247793.s002.pdf]

PLOS ONE

Reduced soil fauna decomposition in a high background radiation area

--Manuscript Draft--

Manuscript Number:	PONE-D-20-38999
Article Type:	Research Article
Full Title:	Reduced soil fauna decomposition in a high background radiation area
Short Title:	Reduced soil fauna decomposition in a high background radiation area
Corresponding Author:	Hallvard Haanes<br>Norwegian Radiation and Nuclear Safety Authority<br>Østerås, Bærum NORWAY
Keywords:	litter decomposition; soil meiofauna activity; litterbags; radioactivity; thorium decay series
Abstract:	Decomposition of litter and organic matter is a very important soil ecosystem function where soil fauna play an important role. Knowledge of the responses in decomposition and soil fauna to different stressors is therefore crucial. However, the extent to which radioactivity affect soil fauna is not so well known. There are some results showing effects at uranium mines but results on effects of anthropogenic radionuclides on soil fauna decomposition in Chernobyl are contradictory. We hypothesize that naturally occurring radionuclides affect soil fauna and thus litter decomposition, which will covary with radionuclide levels when accounting for important soil parameters. We have therefore used standardised litterbags with two different mesh sizes filled with birch leaves (*Betula pubescens*) to assess litter decomposition in an area with enhanced levels of naturally occurring radionuclides in the thorium (^{232}Th) and uranium (^{238}U) decay chains while controlling for variation in important soil parameters like pH, organic matter content, moisture and large grain size. We show that decomposition rate is higher in litterbags with large mesh size compared to litterbags with a fine mesh size that excludes soil fauna. We also find that litter dried at room temperature is decomposed at a faster rate than litter dried in oven (60 ^{0}C). This was surprising given the associated denaturation of proteins and anticipated increased nutritional level but may be explained by the increased stiffness of oven-dried litter. This result is important since different studies often use either oven-dried or room temperature-dried litter. Taking the above into account, we explore statistical models to show large and expected effects of soil parameters but also significant effects on litter decomposition of the naturally occurring radionuclide levels. We use the ERICA tool to estimate total dose rate per coarse litterbag for four different model organisms, and in subsequent different statistical models we identify that the model including the dose rates of a small tube-shape is the best statistical model. In another statistical model including soil parameters and radionuclide distributions, ^{226}Ra (or uranium precursory radionuclides) explain variation in litter decomposition while ^{228}Ra (and precursors) do not. This may hint to chemical toxicity effects of uranium. However, when combining this model with the best model, the resulting simplified model is equal to the tube-shape dose-rate model. There is thus a need for more research on how naturally occurring radionuclides affect soil fauna, but the study at hand show the importance of an ecosystem approach and the ecosystem parameter soil decomposition.
Order of Authors:	Hallvard Haanes<br>Haanes Gjelsvik
Additional Information:	
**Question**	**Response**
**Financial Disclosure**<br><br>Enter a financial disclosure statement that describes the sources of funding for the work included in this submission. Review	NO - The funders had no role in study design, data collection and analysis, decision to publish, or preparation of the manuscript.

Powered by Editorial Manager® and ProduXion Manager® from Aries Systems Corporation

the submission guidelines for detailed requirements. View published research articles from *PLOS ONE* for specific examples.<br><br>This statement is required for submission and **will appear in the published article** if the submission is accepted. Please make sure it is accurate.<br><br>**Unfunded studies**<br>Enter: *The author(s) received no specific funding for this work.*<br><br>**Funded studies**<br>Enter a statement with the following details:<br>• Initials of the authors who received each award<br>• Grant numbers awarded to each author<br>• The full name of each funder<br>• URL of each funder website<br>• Did the sponsors or funders play any role in the study design, data collection and analysis, decision to publish, or preparation of the manuscript?<br>• **NO** - Include this sentence at the end of your statement: *The funders had no role in study design, data collection and analysis, decision to publish, or preparation of the manuscript.*<br>• **YES** - Specify the role(s) played.<br><br>* typeset	
**Competing Interests**<br><br>Use the instructions below to enter a competing interest statement for this submission. On behalf of all authors, disclose any competing interests that could be perceived to bias this work—acknowledging all financial support and any other relevant financial or non-financial competing interests.<br><br>This statement **will appear in the published article** if the submission is accepted. Please make sure it is accurate. View published research articles from *PLOS ONE* for specific examples.	he authors have declared that no competing interests exist

**NO authors have competing interests**<br><br>Enter: *The authors have declared that no competing interests exist.*<br><br>**Authors with competing interests**<br><br>Enter competing interest details beginning with this statement:<br><br>*I have read the journal's policy and the authors of this manuscript have the following competing interests: [insert competing interests here]*<br><br>* typeset	
**Ethics Statement**<br><br>Enter an ethics statement for this submission. This statement is required if the study involved:<br><br>• Human participants<br>• Human specimens or tissue<br>• Vertebrate animals or cephalopods<br>• Vertebrate embryos or tissues<br>• Field research<br><br>Write "N/A" if the submission does not require an ethics statement.<br><br>General guidance is provided below. Consult the submission guidelines for detailed instructions. **Make sure that all information entered here is included in the Methods section of the manuscript.**	N/A

Powered by Editorial Manager® and ProduXion Manager® from Aries Systems Corporation

**Format for specific study types**<br><br>**Human Subject Research (involving human participants and/or tissue)**<br>• Give the name of the institutional review board or ethics committee that approved the study<br>• Include the approval number and/or a statement indicating approval of this research<br>• Indicate the form of consent obtained (written/oral) or the reason that consent was not obtained (e.g. the data were analyzed anonymously)<br><br>**Animal Research (involving vertebrate animals, embryos or tissues)**<br>• Provide the name of the Institutional Animal Care and Use Committee (IACUC) or other relevant ethics board that reviewed the study protocol, and indicate whether they approved this research or granted a formal waiver of ethical approval<br>• Include an approval number if one was obtained<br>• If the study involved *non-human primates*, add *additional details* about animal welfare and steps taken to ameliorate suffering<br>• If anesthesia, euthanasia, or any kind of animal sacrifice is part of the study, include briefly which substances and/or methods were applied<br><br>**Field Research**<br><br>Include the following details if this study involves the collection of plant, animal, or other materials from a natural setting:<br>• Field permit number<br>• Name of the institution or relevant body that granted permission	
**Data Availability**<br><br>Authors are required to make all data underlying the findings described fully available, without restriction, and from the time of publication. PLOS allows rare exceptions to address legal and ethical concerns. See the PLOS Data Policy and FAQ for detailed information.	Yes - all data are fully available without restriction

A Data Availability Statement describing where the data can be found is required at submission. Your answers to this question constitute the Data Availability Statement and **will be published in the article**, if accepted.<br><br>**Important:** Stating 'data available on request from the author' is not sufficient. If your data are only available upon request, select 'No' for the first question and explain your exceptional situation in the text box.<br><br>Do the authors confirm that all data underlying the findings described in their manuscript are fully available without restriction?	
**Describe where the data may be found in full sentences. If you are copying our sample text, replace any instances of XXX with the appropriate details.**<br><br>• If the data are **held or will be held in a public repository**, include URLs, accession numbers or DOIs. If this information will only be available after acceptance, indicate this by ticking the box below. For example: *All XXX files are available from the XXX database (accession number(s) XXX, XXX.).*<br>• If the data are all contained **within the manuscript and/or Supporting Information files**, enter the following: *All relevant data are within the manuscript and its Supporting Information files.*<br>• If neither of these applies but you are able to provide **details of access elsewhere**, with or without limitations, please do so. For example:<br><br>*Data cannot be shared publicly because of [XXX]. Data are available from the XXX Institutional Data Access / Ethics Committee (contact via XXX) for researchers who meet the criteria for access to confidential data.*<br><br>*The data underlying the results presented in the study are available from (include the name of the third party*	The data underlying the results presented in the study will be made available as cvs of all data before publication from the Mendely data sharing: https://data.mendeley.com/datasets

Powered by Editorial Manager® and ProduXion Manager® from Aries Systems Corporation

and contact information or URL).<br>• This text is appropriate if the data are owned by a third party and authors do not have permission to share the data.<br><br>* typeset	
Additional data availability information:	Tick here if the URLs/accession numbers/DOIs will be available only after acceptance of the manuscript for publication so that we can ensure their inclusion before publication.

Powered by Editorial Manager® and ProduXion Manager® from Aries Systems Corporation

Click here to access/download;Manuscript;Haanes_Gjelsvik_MS_litterbag.d

Reduced soil fauna decomposition in a high background radiation area

Hallvard Haanes[a, b, *], Runhild Gjelsvik[a, b]

a) Norwegian Radiation and Nuclear Safety Authority, P. O. Box 329 Skøyen NO-0213 Oslo

b) Centre for Environmental Radioactivity (CERAD CoE), P.O. box 5003, NO-1432 Ås, Norway

Corresponding author*email; Hallvard.Haanes@dsa.no

Authors contributed equally.

Keywords; litter decomposition, soil meiofauna activity, litterbags, radioactivity, thorium decay series

Abstract

Decomposition of litter and organic matter is a very important soil ecosystem function where soil fauna play an important role. Knowledge of the responses in decomposition and soil fauna to different stressors is therefore crucial. However, the extent to which radioactivity affect soil fauna is not so well known. There are some results showing effects at uranium mines but results on effects of anthropogenic radionuclides on soil fauna decomposition in Chernobyl are contradictory. We hypothesize that naturally occurring radionuclides affect soil fauna and thus litter decomposition, which will covary with radionuclide levels when accounting for important soil parameters. We have therefore used standardised litterbags with two different mesh sizes filled with birch leaves (*Betula pubescens*) to assess litter decomposition in an area with enhanced levels of naturally occurring radionuclides in the thorium (^{232}Th) and uranium (^{238}U) decay chains while controlling for variation in important soil parameters like pH, organic matter content, moisture and large grain size. We show that decomposition rate is higher in litterbags with large mesh size compared to litterbags with a fine mesh size that excludes soil fauna. We also find that litter dried at room temperature is decomposed at a faster rate than litter dried in oven (60°C). This was surprising given the associated denaturation of proteins and anticipated increased nutritional level but may be explained by the increased stiffness of oven-dried litter. This result is important since different studies often use either oven-dried or room temperature-dried litter. Taking the above into account, we explore statistical models to show large and expected effects of soil parameters but also significant effects on litter decomposition of the naturally occurring radionuclide levels. We use the ERICA tool to estimate total dose rate per coarse litterbag for four different model organisms, and in subsequent different statistical models we identify that the model including the dose rates of a small tube-shape is the best statistical model. In another statistical model including soil parameters and radionuclide distributions, ^{226}Ra (or uranium precursory radionuclides) explain variation in litter decomposition while ^{228}Ra (and precursors) do not. This may hint to chemical toxicity effects of uranium. However, when combining this model with the best model, the resulting simplified model is equal to the tube-shape dose-rate model. There is thus a need for more research on how naturally occurring radionuclides affect soil fauna, but the study at hand show the importance of an ecosystem approach and the ecosystem parameter soil decomposition.

1. Introduction

Soils provide important ecosystem functions and services like decomposition, nutrient cycling, nitrogen fixation, control of greenhouse gases/CO_2 fluxes, food production and agriculture [1-3]. An important part is their soil fauna, which often is very species rich and links to above-ground biodiversity [4,5] and land productivity [2,3]. Soil organisms are important for soil formation and regulate major ecosystem processes like organic matter turnover, nutrient cycling and mineralization, affecting both microbes and plants [6]. Soil fauna affect soil microbes and mineralisation directly by selective feeding, which alters microbe activity, abundance and community, and indirectly through increasing food availability by fragmenting and mixing organic matter in soil [6]. Organic matter is then broken down by microbes which releases nutrients directly to soil or indirectly through symbionts or predators' extrection (nematodes and collembola). This is called mineralization and is of key importance at ecosystem scale because it determines the nutrient availabilty for plant uptake and productivity [6-11].

Knowledge of the responses of soil ecosystems and faunas to different stressors is therefore crucial to mankind to identify any threats to these important ecosystem services. Assessment of ecosystem responses is however difficult due to their complexity and how functions relate to species richness and interactions among species and food webs [12]. Ecosystem responses can for example not be predicted as the sum of responses in each of the constituent species [13]. Neither ecosystem-level parameters nor indirect effects can be assessed through single species experiments [14,15]. Examples of important parameters are biodiversity and ecosystem function, which are linked [16,17]. In soils this includes decomposition and mineralization, which influence land productivity [2].

Litterbag field experiments have shown that soil fauna is important to both decomposition and mineralization rates, depending on climate and litter quality with regard to N:C and lignin content [18-24]. Unambiguous effects have been found in deciduous forest [23], and for birch in different

ecosystems and climates [21,25]. Soil fauna can be classified according to their diameter, which is smaller than 100 µm for microflora and microfauna taxa like Bacteria, Fungi, Protozoa and Nematoda, and smaller than 2 mm for mesofauna taxa like Collembola (springtails), Acari (mites), and Enchytraeidae while macrofauna taxa like Lumbricina (earthworms), Isopoda and Mollusca have a wider width [6,26,27]. It has been show that decomposition in litterbags with different mesh sizes may be affected by differences in microclimate, moisture and aeration relating to mesh size difference that may involve a more efficient micro-fauna/flora decomposition, except in very dry or wet conditions [18]. Depending on mesh size, different sized soil fauna can thus enter and affect decomposition [24] and a combination of litterbags with different mesh sizes can be used to assess the decomposing effects of meso and macro soil fauna compared to the effects of microbes and fungus only [28]. Soil fauna activity, abundance and diversity are often negatively affected by stressors like chemicals [29-32], and litterbags have been used to both verify deleterious effects of anthropogenic radioactive pollution [33], as well as contradict them [34].

Negative effects of radioactivity on soil fauna have been extensively reported from Russian spill sites like Chernobyl but with different sensitivities among different soil taxa with earthworms, millipeds, collembolans and mites being most appropriate as bioindicators [35]. Since these taxa in addition to Enchytraeids are important to decomposition [18,36], Chernobyl litterbag results showing reduced decomposition [33] does not seem surprising, while other Chernobyl litterbag results which show the opposite and no effect from radioactivity [34] are somewhat surprising. Effects on soil fauna have also been demonstrated using litterbags and bait lamina in Portugal at uranium contaminated mining sites [37,38]. As far as we know, nobody has investigated for effects on soil fauna from naturally occurring radioactivity. We hypothesize that naturally occurring radionuclides will affect soil fauna so that litter decomposition will covary with radionuclide levels when accounting for important soil parameters. To address this, we used a combination of litterbags with a fine or a coarse mesh to

assess litter decomposition along a gradient of naturally occurring radioactivity within an area with elevated background radiation, the Fen igneous complex [39].

2. Materials and Methods

2.1 Area description

The Fen complex (59.2756°N, 9.3110°E) is the footprint of an eroded volcano (580 mill y) consisting of calcareous carbonatite bedrock types with naturally enhanced radioactivity (Fig. 1). One part, Mining hill, consists of red rock with activity concentrations from 670 to 12000 Bq kg^{-1} of ^{232}Th and from 44 to 550 Bq kg^{-1} of ^{238}U [40]. Here, bedrock comes to the surface and within Mining hill there are legacy mines and deposits of waste rock. The soil here have much higher than normal levels of the thorium (^{232}Th) series radionuclides [41,42], even though there is large spatial heterogeneity in the distribution of radionuclides [43]. This variation makes Mining hill a suitable place for assessment of soil ecosystem responses through field experiments. It is forested with pine (*Pinus sylvestris*) stands and birch (*Betula pubescens*) is common. Moreover, radionuclides in the soil are known to transfer to earthworms [44]. Also, in Mining hill, outdoor levels of thoron (^{220}Rn) and progenies are several orders higher than normal due to both mine ventilation, waste rock deposits [45] and soil exhalation [39], suggesting significant levels in soil gas.

2.2 Litterbags, litter treatment and standardisation

To perform a field experiment within Mining hill, we prepared 100 litterbags. Half of these were made from two layers fine-meshed (0.1 mm) and the other half from coarse-meshed (2 mm) nylon (Elko filtering co) by gluing together the edges of rectangles of one mesh size with Marin & Teknik (Casco; Supplementary), forming an inner size of 15 x 20 cm. The coarse-meshed litterbags were suited with Velcro for easy opening while the fine meshed bags were glued together after filling with litter to ensure no mesofauna penetration.

Litterbag studies have shown that different quality types of litter with different N:C ratios are decomposed differently [28]. We used standardised amounts (d.w) of newly naturally shed leaves from birch (*Betula pubescens*), which is present at each of the experimental localities. This birch species has C:N ratios ranging from 20 to 33 across Europe [46], which is the C:N ratio range that is most affected by soil fauna decomposition effects [23]. Freshly abscised leaves were sampled in an area with normal background radioactivity during litter-fall on September 28 from a regularly maintained lawn. Low levels of radionuclides associated with the sampled leaves were verified for two subsamples by gamma spectrometry (HPGe).

Other studies of litter decomposition have used litter dried at either at room temperature or by higher temperatures in oven. We hypothesize that heat treatment will involve different nutrient availability, which will increase decomposition rate. To assess this, we dried half of the leaves on newspapers at room temperature for five days, while the other half was dried in aluminium trays within paper bags in an oven at 60 °C for 48 hours. This is a commonly applied temperature which involves maximum water removal but also denaturation of one third of leaf proteins [47]. We wanted to assess whether oven-dried litter is decomposed / lost from litterbags faster than litter dried at room temperature. The two forms of dried litter were therefore applied in the same amount in a balanced experimental design. Afterwards, the fine mesh-sized litterbags were filled with 7.99-8.03 grams of dried litter (mean: 8.00, SD: 0.01), and the coarse-sized litterbags were filled with from 7.77 to 8.00 grams of dried litter (mean:7.86, SD:0.05, Fig. 2). The small but significant ($t=22$, $p<<0.01$) difference in litter fill was due to loss of small fragments through the meshes during filling.

2.3 Experimental Design

Within Mining hill, five localities were established through gamma spectrometry to represent a gradient of soil radionuclides in the ^{232}Th decay chain. Each locality consisted of a 5x5 meter square within a pine stand of approximately the same height and density. Twenty large litterbags were

placed at each locality, two by two into quartets of four litterbags, with five quartets per locality at the centre and corners of the locality square, except at one location (Loc26). At Loc26, one quartet was placed at another location within the square and another quartet was placed right outside the square, and four litterbags in one of the corners were placed four by one (next to each other) due to rocks and very uneven substrate. The pattern of litterbags across localities were situated according to litterbag treatment to achieve a balanced random experimental design. Each quartet consisted of two fine-meshed and two coarse-meshed, each of which contained one litterbag with air-dried and one with oven-dried litter. Fine and coarse litterbags always lay side by side, but elsewise placements within each quartet was random. Within quartets, distances were the same, with diagonal litterbags (centres) separated by 0.4 meters and adjacent ones (centres) separated by 0.2 meters. Within localities, the quartet centres were separated by 1.4 to 5.7 meters (mean: 3.6, SD:1.1). Within the whole study area, the centres of localities were separated by 35 to 340 meters (mean: 180, SD:115). Litterbags positions therefore represents three different spatial scale levels.

All litterbags were deployed exactly a year from October 17, which correspond with the period when litterfall ends at this altitude and latitude. After deployment the litter samples were dried in oven at 105 ^{o} C for 24 h and the difference in dry litter weight before and after deployment was used to calculate the amount (grams) of litter loss per litter bag. Since variation among litterbags in initial litter mass was very small and deployment period was equal, the mass loss was used directly in analyses rather than lost fraction of litter per litterbag.

2.4 Soil sampling and analyses

To be able to account for spatial heterogeneity, soil core (Ø=5.4 cm with an entrance of 4.8 cm) samples were taken beneath each of the coarse litterbags (0-6 cm) per litterbag quartet. Soil samples varied in wet weight from 36.4 to 138.5 g (mean:86.2, SD:26.9). Each soil sample was dried at 105 ^{o}C for 24 hours, and the mass difference between wet and dry weight of each sample was used to

assess its fraction of soil moisture. After drying, samples were sieved (2 mm) and soil particles larger than 2 mm were retained to calculate their fraction of the samples total mass. From the sieved soil (<2 mm), subsamples were taken for assessment of pH, organic matter content and activity concentrations of radionuclides. Soil pH was measured using an inoLab 7110 pH meter XXX () according to ISO TC WI: 2003 (CEN/BT/Task Force 151) using a buffer to counter effects of ionic bindings. Levels of ^{232}Th and ^{238}U decay series radionuclides in soil samples were assessed through gamma spectrometry using high purity Germanium (HPGe) detectors analysing for ^{228}Ra (through ^{228}Ac at the 338, 911 and 969 KeV energies weighted their uncertainties), ^{226}Ra (through uncertainty-weighted ^{222}Rn daughters ^{214}Pb at energies 295 and 352 KeV and ^{214}Bi at 609 KeV), assuming approximate secular equilibrium for subsequent progeny except ^{210}Pb and ^{210}Po. Significant amounts of these latter radionuclides can due to the long half-life of ^{210}Pb deposit from air and ^{210}Pb was therefore analysed separately (at energy KeV with a point-source correction for density with regard to the high OM content of some samples), and secular equilibrium was assumed for its progeny ^{210}Po. Due to a limited number of such detectors, only 20 soil samples were analysed for ^{210}Pb across localities. In addition, soil samples were analysed for ^{137}Cs (at energy 661.65 KeV). These HPGe detectors have relative efficiencies of 23% to 50% and cover energies from 20 keV to 3000 keV. They are placed in a low background laboratory and are regularly controlled against a traceable source. Each analysed sample was placed within a circular plastic measurement geometry (36.4 mL), and results corrected for decay since sampling. Within each geometry, the density of each sample was calculated as mass (kg d.w. soil) per volume (36 mL). Finally, organic matter (OM) content was measured by loss on ignition in an oven, using 5 hours to reach and 12 hours at 550 ^{0}C, as the difference in mass before and after ignition compared to the dry weight before ignition.

2.5 DOSIMETRY OF SOIL FAUNA

The ERICA tool [48] was used for dosimetry, modelling organisms in a terrestrial ecosystem with 100% occupancy below ground with the measured soil samples of ^{226}Ra, ^{228}Ra and Cs137 as input. The measurements of these radionuclides were used as proxy for all the modelled radionuclides, assuming secular equilibrium. In the newest version of the ERICA tool, the contribution of radionuclides in the thorium and uranium decay chains with less than 10 days decay are included in the dosimetry of the previous radionuclide exceeding 10 days half-life [48]. Three radionuclides in the ^{232}Th decay chain and seven radionuclides in the ^{238}U decay chain were therefore included in the ERICA modelling, as well as the measured levels of ^{137}Cs. After ERICA assessment, external and total dose rate was summed up across radionuclides per soil sample.

For this assessment, the default ERICA annelid (5.2 gram, Ksi: 0.1, Chi: 0.1) and detritivore arthropod (0.4 gram, Ksi: 0.35, Chi: 0.18) were modelled, as was also two smaller shapes that better represent the soil mesofauna: a smaller tube (0.4 gram, Ksi: 0.08, Chi: 0.08) and a small box (0.4 gram, Ksi: 0.4, Chi: 0.4). The mass range allowed in ERICA spans 1.7x10E-4 kg to 6.6 kg, and assuming a 70 % water content, this involves minimum dimensions that are 4.5 x 4.5 x 11.3 mm for a rectangular box, or 1.2 x 1.2 x 16 mm for a tube shape. These shapes are similar in shape but larger than a typical Collembola or Enchytraeid. Collembola range from 0.13 to 2 mm in width and 0.16 to 6 mm in length, while Enchytraeids range from 0.5 to 1.3 mm in width and 1 to 40 mm in length and earthworms range from 2 to 20 mm in with and 12 to 80 mm [6,26,27].

In ERICA tool, the concentration (CR) ratio equals the ratio between activity concentration per wet weight of the organism divided by the activity concentration per dry weight soil. However, there are few data on the CR of small organisms living on or within soil, or their typical water content so that CR could be calculated from transfer factors (TF), which is the ratio between activity concentrations of organisms and soil both per dry weight.

For the annelid, ERICA database CR values were used for all radionuclides except the radium isotopes where the default CR values for similar reference organism are the only choice. For enchytraeids CR's are very scarce and there are no suggestions in ERICA. Among earthworm species, CR's for thorium isotopes vary from 0.006 to 0.024 while uranium CR's range from 0.005 to 0.064 [44,49,50]. In ERICA tool, the annelid default CR is 0.009 for thorium isotopes and 0.034 for uranium isotopes. For the smaller tube-shape to represent Enchytraeids, we used literature-based CR's of 0.015 for the thorium isotopes and 0.035 for the uranium isotopes. CR's for lead and radium isotopes are scarce but for one of the earthworm species above, *Eisenia andrei* (50-70 mm length), assuming 80% water content, these values are 0.4 and 0.82, respectively [49]. For the earthworm genus Lumbricina*,* CR's of 0.095 and 0.096 have been reported for ^{210}Pb and ^{210}Po [50]. In ERICA, the default annelid CR's are 0.043, 0.48 and 0.01 for ^{226}Ra, ^{210}Pb and ^{210}Po. For the small tube-shape (Enchytraeid), we used CR's of 0.48 for ^{210}Pb and 0.01 for ^{210}Po (similar between ERICA and literature), and 0.08 for ^{137}Cs (as ERICA annelid CR). However, the for the tube-shape we used a literature-based CR of 0.8 for the radium isotopes.

For the detritivore arthropod ERICA database CR values were used for all radionuclides except where missing, as for the polonium isotope where the default CR from a similar reference organism was used and the thorium CR where the default combination method was used. The ERICA detrivore arthropod default CR's are 0.005 for thorium isotopes, 0.01 for uranium isotopes, 0.04 for ^{210}Pb, 0.01 for ^{210}Po and 0.11 for ^{137}Cs. For woodlice and diplopods, literature ^{226}Ra CR ranges from 0.68 to 1.2 and from 0.54 to 0.79, respectively, when assuming 65% water content [51]. Woodlice have great transpiration power but the water content is typically around 65% for soil arthropods like diplopods and Collembola [50,52]. For the box shape representing Collembola (and Acari), we therefore used the ERICA CR values for all radionuclides except for the radium isotopes where we used a CR of 0.66 (average diplopod CR).

2.6 STATISTICAL ANALYSES AND MAPS

The fraction of soil fragments >2mm, soil OM, pH and ^{137}Cs levels, as well as litter loss had an approximate lognormal distribution. Similarly, naturally occurring radionuclides in general have lognormal distributions [53,54], as have also been shown for the soil ^{232}Th progeny in the study area [55,56]. These parameters were therefore log transformed ($\log_{10} x + 0.001$) prior to statistical analyses. It has been highlighted to assess actual weight loss over time and not percent [18]. This especially applies if initial weight or deployment period varies. Since the same litter mass was used to fill each litterbag (but see section 2.2 with regard to a slight loss of litter from coarse mesh bags) and the deployment period was the same for all litterbags, we use lost litter (grams d.w.) rather than rate of loss in our statistical analyses.

To assess spatial autocorrelation, correlations were made between a) an array of all possible pairwise physical distances between all soil samples (n=1225) and b) each of arrays of the corresponding pairwise absolute differences between each of these soil sample pairs for each of the soil parameters. The distance array was calculated using the dist() function since localities are separated at most by 100's of meters, while the corresponding pairwise parameter differences were calculated with the combn() function, both in base R [57] using Euclidean distances. In addition, range, mean and variation was calculated for the pairwise differences in each soil parameter separately for each spatial scale level, i.e. for litterbag pairs separated by 20-40 cm, pairs separated by 1.4 to 5.7 m, and for pairs separated by 35 to 340 m.

Statistical analyses were done in R [57]. To assess what may explain litter loss, additive linear models of the litter loss ($\log_{10}$ transformed) among litterbags were explored, including as explanatory variables whether litter was dried in oven or room temperature, the soil parameters (chapter 2.4, $\log_{10}$ transformed) and either activity concentration distributions ($\log_{10}$ transformed) of the radionuclides most important to dose rates, or the dose rates ($\log_{10}$ transformed) estimated at each

coarse litterbag through the ERICA tool for either of the four assessed organism types. Each model was simplified removing non-significant terms (potentially explanatory variables), one at a time. Remaining models were compared through Aikakes information criterion (AIC) penalized according to small sample size and number of parameters assessed [58].

Map data was downloaded from the Geological Survey of Norway (NGU) as layers of bedrock (http://geo.ngu.no/kart/berggrunn/) and Holocene deposition (http://geo.ngu.no/kart/losmasse/).

3. Results

3.1 Soil samples

The soil samples from Mining hill contained fragments larger than 2 mm (sieved off) in a fraction that ranged from less than 0.01 to 0.44 (mean: 0.11, median: 0.08, SD: 0.10), but with some variation among the localities (Fig. 3). Soil pH (measured in buffer) ranged from 3.3 to 5.5 (mean: 4.8, median: 4.9, SD: 0.5), with especially one locality standing out (Fig. 4). The fraction of organic matter (OM) content (after drying and sieving) ranged from 0.10 to 0.69 (mean: 0.30, median: 0.32, SD: 0.11), also with substantial variation among localities (Fig. 5). The soil moisture fraction ranged from 0.29 to 0.55 (mean: 0.38, SD: 0.07), involving similar variation among localities (Fig. 6), and had moderate positive correlation to $\log_{10}$-transformed OM ($r=0.63$, $p<<0.01$). Prepared in fixed volumes (36.4 mL) for gamma spectrometry the samples weighed from 14 to 34 grams (d.w.), which was strongly negatively correlated to $\log_{10}$-transformed OM ($r=-0.96$, $p<<0.01$) and involved densities from 0.39 to 0.95 grams/mL (mean: 0.64, SD: 0.16). The content of ^{228}Ra ranged from 470 to 5200 Bq kg^{-1} soil dry weight (mean: 2400, median: 2700, SD: 1400), with much variation and little overlap between localities (Fig. 7). Levels of ^{226}Ra ranged from 24 to 120 Bq kg^{-1} soil dry weight (mean: 45, median: 32, SD: 24), with some variation among localities and especially much within locations 26, 30 and 32 (Fig. 8). Levels of ^{210}Pb (n=20) were for all except one locality higher than ^{226}Ra levels (Fig. 9), and for

samples with no analysed ^{210}Pb, the location-wise ratio of ^{210}Pb to ^{226}Ra was used to estimate ^{210}Pb from the analysed ^{226}Ra value.

The $\log_{10}$-transformed ^{228}Ra levels were weakly correlated to $\log_{10}$-transformed levels of ^{226}Ra ($r=0.41$, $p<0.01$), negatively weakly correlated to $\log_{10}$-transformed OM ($r=-0.36$, $p<0.02$) but not to $\log_{10}$-transformed soil pH ($r=-0.13$, $p>0.4$). The $\log_{10}$-transformed ^{226}Ra was also weakly negatively correlated to $\log_{10}$-transformed OM ($r=-0.29$, $p<0.05$) but neither to $\log_{10}$-transformed soil pH ($r=0.09$, $p>0.5$). Log_{10} transformed soil particle fraction >2mm was negatively weakly correlated to $\log_{10}$ transformed ^{226}Ra ($r=-0.31$, $p<0.05$) and almost to ^{228}Ra ($r=-0.26$, $p=0.07$). Soil levels of ^{137}Cs ranged from 15 to 120 Bq kg^{-1} soil dry weight (mean: 35, SD: 19), also with considerable variation among localities but at very low levels (Fig. 10).

Regarding spatial autocorrelation, all soil parameters had significant but at varying strengths correlations to the physical distances between pairs of soil samples, while litter loss itself did not (Table 1). This included a weak negative spatial autocorrelation for $\log_{10}$ of soil pH and a weak positive spatial autocorrelation for $\log_{10}$ of both soil ^{228}Ra and soil ^{226}Ra, while no spatial autocorrelation at all for litter loss (only coarse litterbags). The pattern is also clear among the three levels of spatial scales, as the means and standard deviations of soil ^{228}Ra pairwise absolute differences increase by doubling and tripling, respectively, with spatial scale (from within quartets, to within locations and within study area, Table 2). By comparison, the mean and standard deviation of pairwise absolute difference of soil ^{226}Ra also increased from within quartets to within localities but not from within localities to within the study area, expressing spatial autocorrelation but a less clear gradient among localities. The ^{137}Cs absolute differences between soil samples have similar means and standard deviations between the three scale levels (Table 2).

3.2 Dosimetry

External dose rates estimated for the default annelid with ERICA ranged from 0.7 to 6.8 µGy h^{-1} (mean: 3.1, SD: 1.8) with clear differences between the localities (Fig. 11), and were very similar in the other three modelled organisms, differing maximally by 0.016 µGy h^{-1} for any radionuclide or on average only 0.001 µGy h^{-1} across radionuclides. Including internal dose rates, the total dose rates varied between the four modelled organisms and ranged from 1.8 to 17 µGy h^{-1} (mean: 8.2, SD: 4.6) for the annelid, from 1.4 to 13 µGy h^{-1} (mean: 6.1, SD: 3.4) for the detrivore arthropod, from 5.4 to 32 µGy h^{-1} (mean: 16, SD: 8.0) for the small tube shape and from 3.8 to 20 µGy h^{-1} (mean: 10, SD: 4.9) for the box shape. The pattern across localities was relatively similar for the four organisms (Fig. 12) but with higher levels and more variation in the two additional shapes. Among radionuclides, the main contributions to dose rate came from the radionuclides in the ^{232}Th chain and from ^{226}Ra (Table 3). Differences between the four organisms in the radium isotopes are due to the different applied CR's.

3.3 Litter loss

Within Mining hill, large litter bags had lost from 2.5 to 6.1 grams (d.w.) litter (mean: 3.6, SD: 0.7) after one year of deployment during the field experiment. There were clear differences in litter loss between coarse and fine meshed litterbags (t=8, p<<0.01), as well as between litterbags with litter dried in oven and room temperature (t=3, p<0.01, Fig. 13). The coarse meshed litterbags lost from 2.5 to 6.1 grams of litter (d.w., loss mean: 4.0, SD: 0.8), while the fine meshed litterbags lost from 2.6 to 4.5 grams litter (d.w., loss mean: 3.1, SD: 0.4). Within quartets, pairs of coarse meshed litterbags with air-dried and oven-dried litter differed with 0.1 to 2.5 grams in loss of litter (d.w., loss mean: 0.7, SD: 0.6), while the corresponding fine meshed litterbag pairs with air-dried and oven-dried litter within quartets differed with 0.1 to 1.3 grams in loss of litter (d.w., loss mean: 0.4, SD: 0.3). By comparison, within quartets, pairs of litterbags with oven-dried litter with either coarse or fine mesh

differed with from 0.1 to 1.7 grams litter loss (d.w., loss mean: 0.8, SD: 0.4) while pairs of litterbags with air-dried litter with either coarse or fine mesh differed with from 0.1 to 2.6 grams of litter loss (d.w., loss mean: 1.1, SD: 0.7). Differences between litterbag litter loss was thus significant within quartets (relating to mesh size and drying regime) but larger among quartets and localities.

For the linear statistical model of coarse meshed litterbag loss including radionuclide distributions, the radium isotopes were chosen since these contribute most to dose rates and covary with those other radionuclides that also contribute significantly (Table 3). For this model (adj R^2=0.40, F(36, 7)=5.2, p<0.001), non-significant terms were moisture (t=1.3, p<0.21), pH (log_{10} transformed, t=-1.6, p<0.12) and surprisingly ^{228}Ra (log_{10} transformed, t=-1.3, p<0.19). Since simplification of this model only included ^{226}Ra and not ^{228}Ra, and since much dose contribution is from the ^{232}Th chain (Table 3), an alternative model including only ^{228}Ra was therefore also subsequently explored through AIC. For the four linear statistical models of litter loss including the estimated dose rates of organisms types (adj R^2=0.33-0.40, F(36, 7)=4.5-6.0, p<0.002), non-significant terms included moist (t=1.2-1.3, p>0.21) for all models while size particle fraction > 2mm (log_{10} transformed) was non-significant (t=-1.6, p>0.11) for the two default organism models, nearly significant for the small tube-shape (t=-2.0, p<0.052) and significant for the box shape (t=-2.2, p<0.04). After model simplification, comparison of the six explored models show that their ability to explain variation in loss from litterbags (log_{10} transformed) is relatively similar but that the two best models are the one including the dose rate of the tubes-shaped organism and the model including the ^{226}Ra distribution (Table 4). For all six models, the effect of all significant parameters was negative and relatively similar. Among parameter estimates the effect is clearly larger for the soil parameters organic matter fraction (OM) and pH (both log_{10} transformed) but somewhat less for $log_{10}{}^{226}$Ra and especially the dose rate (Table 5). By comparison, the parameter estimate of $log_{10}{}^{228}$Ra was even lower (β=-0.07 SE=0.04). Combining the two best models into one model yielded the non-significant terms $log_{10}{}^{226}$Ra (t=-1.6.0, p<0.128),

log_{10}pH (t=-1.6.0, p<0.122), log_{10}tubeshapeDR (t=-1.7.0, p<0.103) and log_{10}>2mm (t=-2.0, p<0.056), and ended after model simplification again with the best dose rate model (tubeshape DR).

4. Discussion

As expected, there was a much larger loss of litter from litterbags with a coarse mesh size (2 mm) than from litterbags with a fine mesh size (0.1 mm, Figure 2). Presence of macrofaunal like earthworms have a substantial effect on decomposition rate, either directly through litter feeding and/or through indirect effects on micro and meso fauna [36,59], and in a mixed forest ecosystems, earthworms can comsume the entire annual litterfall [6,60]. An effect of the exclusion of soilfauna access to the litter in the fine-meshed litterbags is therefore anticipated. In addition, differences in mesh size may be attributable to differences in microclimate, moisture and aeration that may affect micro-fauna and flora decomposition differently, except in very dry conditions [18]. It is therefore important to assess different mesh sizes and compensate for spatial variation by a high enough spatial resolution when deploying litterbags [22]. However, the direction of the clear effect of the drying regime of the litter among litterbags with both fine and coarse mesh size (Figure 13) and across statistical models was somewhat surprising (Table 5). It is widely known across ecosystems that litter decomposition varies among litter species depending on and increasing with litter quality and levels of C, N and lignin [59,61-63]. Since drying in oven at 60°C involves denaturation of one third of leaf proteins [47], we anticipated an effect relating to an increased decomposition due to nutrients being more available. The opposite direction of the effect may be due to the increased stiffness of litter after oven drying, possibly making the litter less available to soil fauna fragmentation. Since some litterbag studies use only oven dried litter while other studies use room-dried litter, knowledge about the size of this effect is important when such studies are compared.

The most important soil parameters were assessed to avoid confounding factors and showed like radionuclide distributions different variation and gradients across locations and litterbags (Figures 3-

10), involving an ideal opportunity to separate any effects in a statistical analysis. Within the area bedrock is redrock, which is a carbonatite, probably involving less variation in soil parameters than if more bedrock were present. This field experiment can thus with its limited spatial extent be viewed as a common garden experiment, where the design with systematised different distances between litterbags allowed assessment and verification of spatial autocorrelation in the most important parameters. The carbonatite bedrock also explains the relatively high soil pH. Also, the negative effect of soil pH on soil fauna is well known [64]. The fraction of organic matter (OM) observed in the study area is relatively high and probably does not per se involve a negative effect on soil fauna. The negative relationship between litter loss and OM in statistical models demonstrate a higher level of OM beneath litterbags with the lowest degrees of decomposition. This is probably due to a lower abundance or activity of soil fauna, as organic matter will build up when decomposition is low. Finally, and most tantalising, when taking into the account the covariation of important soil parameters, there was a clear statistical effect of the radionuclide levels in this area.

However, the results of the statistical model for radionuclide distribution were surprising given their activity concentrations in the study area and the associated dose rates (Table 3). It was surprising that ^{226}Ra was a highly significant term while ^{228}Ra was not. This strongly hints to other effects on litter decomposition than just dose rates. The sizes of the effect of terms on litter loss also support this notion, with the ^{226}Ra term having an effect around five times larger than both the dose rate terms and the ^{228}Ra term in the statistical models. However, when the two best models were combined and model simplification performed, the subsequent model was the same as the best dose rate model, highlighting an effect of radionuclide dose rate on soil fauna in the area. Interestingly, removal of the ^{226}Ra term resulted in the pH term becoming significant, suggesting these terms explaining similar parts of variation in litter loss.

A potential explanation for the significance of the term on activity concentration of ^{226}Ra but not ^{228}Ra, may be that radionuclide toxicity also is important to soil fauna. Within the study area the

activity concentrations of ^{228}Ra are from 20 to 30 times greater than for ^{226}Ra, while the half-life of ^{226}Ra (1600 y) is 280 times longer than for ^{228}Ra (5.7). By comparison, progenitor radionuclides of ^{226}Ra, uranium isotopes ^{234}U and ^{238}U, have half-lives of 250 ky and 4.5e9. The number of radionuclide atoms present and thus toxicity of these radionuclides with very long half-lives may therefore be important. A recent epidemiological study suggest a higher chemical than radiological risk from ^{226}Ra [65], but it is also well known that uranium has a pronounced chemical toxicity that is more acute than its radiotoxicity [65,66]. Thus, with secular equilibrium for ^{226}Ra with progenitor radionuclides, the large number of uranium atoms and covariation with the distribution of ^{226}Ra could help explain the statistical results.

To be toxic, radionuclides must be bioavailable in aqueous solution and not adsorbed to grain and rock surfaces. The ratio between the amount of a radionuclide in aqueous solution compared to the amount of the radionuclide being adsorbed is known as the distribution coefficient (K_d), which is highly dependent on element, soil-type and soil parameters. For the study area, it has been reported that 77 to 94 % of thorium and 38 to 69 % of uranium present is irreversibly bound to soil, yielding arithmetic mean (AM) K_ds of 5600 (SD:4700) and 280 (SD: 320), respectively, suggesting low availability of thorium but high availability of uranium [41]. By comparison, in loam, sand and organic soils, uranium has some bioavailability with AM K_d values of 2000-3000, while radium is less available in loam and sandy soils with AM K_ds of 8000-15000 but can be very available in organic soils with an AM K_d of 200 [67]. It has been reported that OM has a high adsorption of radium [68], which would involve high ingestion rates of radium for soil organisms eating OM. In this study however, both radium isotopes were weakly and negatively correlated to OM. Adsorption and K_d of radium is also negatively correlated to the concentration of other alkali metals like calcium due to competition for adsorption sites [67], and high soil calcium concentrations can involve radium K_ds as low as 5-100 [69]. Moreover, for uranium, both pH and carbonate are important, with maximal adsorption to soil at $5<pH<7$ but with uranyl aqueous complexes forming at pH above 5 and carbonate complexes that

increase bioavailability, especially at pH above 6 [67,70]. This may be important, remembering the covariation of ^{226}Ra and uranium isotopes (given secular equilibrium) and the seeming overlap between ^{226}Ra and pH in explaining variation in litter loss. Moreover, as could be expected with the redrock in the study area, which consists of hematite-calcite-carbonatite, the cation exchange capacity is very high [41]. With the observed pH up to 5.5 (AM 4.8), and probably high calcium and carbonate levels, adsorption and K_d is probably low for both ^{226}Ra and the uranium isotopes in the study area, highlighting the importance of uranium isotopes. The importance and large impact of uranium on soil fauna and decomposition rates have been demonstrated elsewhere [37,38,71], but there is also a general suggestion for more research on how different soil parameters affect the availability of radium isotopes [67]. Even though the very long half-life and large number of atoms of ^{238}U hints at the importance of uranium toxicity, future research in the study area should assess how different soil parameters here affect both uranium and radium bioavailability.

Whether or not radionuclide dose rates in themselves may affect the litter decomposition of soil fauna has been debated and results from Chernobyl have been contradictory [33,34]. As previously mentioned, our statistical models indicate an explanatory power of dose rate from the naturally occurring radionuclides on variation in litter decomposition but also hints at effects of radionuclide toxicity. In the study at Chernobyl that failed to identify any effects of anthropogenic radionuclides, ^{137}Cs and ^{90}Sr were the dominant ones and assessed dose rates ranged from less than 0.5 to 75 µGy h^{-1} [34], which is well below up to four times as high dose rates as the range in the study at hand. However, stable strontium is not considered toxic [72], and stable caesium at environmental levels is neither toxic to animals, even though both stable and radiocaesium may be somewhat chemically toxic at higher concentrations due to its similarity to kalium [73,74]. Radioceasium is however probably much less chemically toxic than uranium and radium, and in Chernobyl it is probably mainly dose rates that are affecting soil fauna.

Regardless, the study at hand has identified effects on decomposition rate shown by how common soil parameters but also the occurrence of naturally occurring radionuclides explain variation in litter loss from litterbags. Decomposition of OM and litter is an important soil ecosystem function and parameter [6-11], which seems to be affected by changes in soil fauna biodiversity [75,76]. The variation in litter decomposition seen in the study area may be related to soil fauna biodiversity, but this needs to be assessed in future studies. With the effects seen on an ecosystem level in this study, we join present encouragements on more future studies with an ecosystem approach [13,77]. It may be useful to assess different ecosystem parts separately [13], and we suggest to preferentially address effects in earthworms, enchytraeids, collembolas and mites separately. Moreover, in areas like the study area, where soil may be as old as the last ice age, adaptation to radiation may have occurred, and it may be feasible to do irradiation studies of soil fauna extracted both from here and from control sites (where adaptation cannot have occurred since the ice age).

5. Acknowledgements

This work was supported by the Research Council of Norway through its Centres of Excellence funding scheme, project number 223268/F50. This was received thorough the Centre for Environmental Radioactivity, CERAD, Centre of Excellence CoE. We are thankful. We also thank the Geological Survey of Norway (NGU) for use of map data. Thanks to Justin Brown for help with use of ERICA tool and helpful discussions on dosimetry.

6. Tables and figures

Table 1 Correlation strength and significance between physical distance of all pairs of soil samples and corresponding difference (Δ) of each parameter.

Correlation	**Strength (r)**	**Significance (p)**
*Distance (m) * Δ fraction fragments >2mm*	-0.18	<<0.01
*Distance (m) * Δ soil moisture (fraction)*	-0.09	<<0.01
*Distance (m) * Δ log_{10} soil OM (fraction)*	0.08	<<0.01
*Distance (m) * Δ log_{10} soil pH*	-0.34	<<0.01
*Distance (m) * Δ log_{10} soil ^{228}Ra (Bq kg^{-1} d.w.)*	0.37	<<0.01
*Distance (m) * Δ log_{10} soil ^{226}Ra (Bq kg^{-1} d.w.)*	0.31	p<<0.01
*Distance (m) * Δ log_{10} soil ^{137}Cs (Bq kg^{-1} d.w.)*	-0.15	<<0.01
*Distance (m) * Δ litter loss (gram $year^{-1}$)*	0.03	>0.29

Table 2 For ^{228}Ra, ^{226}Ra and ^{137}Cs: range, mean and standard deviation (SD) of absolute differences between all pairs of soil samples within each quartet (smallest spatial scale), within each locality (medium spatial scale), as well as within the whole study area (largest spatial scale).

	**Within quartets**			**Within localities**			**Within study area**		
	Range	Mean	SD	Range	Mean	SD	Range	Mean	SD
Δ Soil ^{228}Ra (Bq kg^{-1} d.w.)	0-1210	250	320	0-3700	610	720	0-4700	1600	1200
Δ Soil ^{226}Ra (Bq kg^{-1} d.w.)	0.8-35	5	7	0-92	18	23	0-97	24	24
Δ Soil ^{137}Cs (Bq kg^{-1} d.w.)	0.1-71	9	14	0-78	11	13	0-103	19	19

Table 3 Range (mean) of dose rates in µGy h^{-1} (external and internal combined) per radionuclide estimated with ERICA tool for the default annelid and detrivore arthropod, as well as for a small tube and box shape representing an Enchytraeid and a woodlouse living within the Fen soil levels.

	**Annelid**	**Arthropod**	**Small tube**	**Small box**
Th-232	0.10-1.1 (0.51)	0.06-0.61 (0.28)	0.16-1.8 (0.83)	0.05-0.60 (0.28)
Ra-228	0.24-2.7 (1.2)	0.24-2.7 (1.2)	0.32-3.6 (1.6)	0.31-3.4 (1.6)
Th-228	1.18-13 (6.0)	0.82-9.0 (4.2)	1.7-19 (8.6)	0.81-9.0 (4.1)
U-238	0.02-0.10 (0.04)	0.01-0.03 (0.01)	0.02-0.10 (0.04)	0.01-0.03 (0.01)
Th-234	0	0	0	0
U-234	0.02-0.11 (0.04)	0.01-0.04 (0.01)	0.02-0.12 (0.04)	0.01-0.03 (0.01)
Th-230	0.01-0.03 (0.01)	0-0.02 (0.01)	0.01-0.05 (0.02)	0-0.02 (0.01)
Ra-226	0.17-0.82 (0.31)	0.17-0.82 (0.31)	2.73-14 (5.0)	2.3-11 (4.2)
Pb-210	0-0.05 (0.01)	0-0.04 (0.01)	0-0.04 (0.01)	0-0.04 (0.01)
Po-210	0.01-0.15 (0.03)	0.01-0.15 (0.03)	0.01-0.14 (0.03)	0.01-0.14 (0.03)
Cs-137	0-0.04 (0.01)	0-0.04 (0.01)	0-0.04 (0.01)	0-0.04 (0.01)

Table 4 Explored simplified linear models ranked according to penalised AIC. Log_{10} transformation was done for fraction of organic matter (OM), pH, fraction of soil particle size>2mm (>2mm), ^{228}Ra, ^{226}Ra, and for the dose rate estimates of each organism type (annelid DR, arthropod DR, tube-shape DR, box-shape DR), not for factorial litter drying regime (dried).

Model terms	Adj R^2	F (df, df)	p-value	AIC
Dried + OM + pH + tube-shape DR	0.36	7.4 (4, 41)	<0.001	-112.8
Dried + OM + ^{226}Ra + >2mm	0.38	7.9 (4, 40)	<0.001	-110.5
Dried + OM + pH + arthropod DR	0.32	6.3 (4, 41)	<0.001	-109.8
Dried + OM + pH + annelid DR	0.32	6.3 (4, 41)	<0.001	-109.6
Dried + OM + ^{228}Ra + >2mm	0.31	6.0 (4, 41)	<0.001	-108.8
Dried + OM + pH + box-shape DR + >2mm	0.40	6.9 (5, 38)	<0.001	-107.1

Table 5 Parameter estimates (β±SE) with the two best simplified linear models of log_{10} litter loss, with log_{10} transformation of fraction of organic matter (OM), pH, fraction of soil particle size>2mm (>2mm), ^{226}Ra and dose rate of organism (tube-shape DR), but not for litter drying regime (dried_oven).

Parameter name	Tube-shape DR model, AIC=-113	226Ra model, AIC=-110
Intercept	1.04 ± 0.17	0.72 ± 0.08
Dried_oven	-0.06 ± 0.02	-0.07 ± 0.02
OM	-0.32 ± 0.07	-0.24 ± 0.06
pH	-0.64 ± 0.24	-
Tubeshape DR	-0.06 ± 0.02	-
^{226}Ra	-	-0.19 ± 0.06
>2mm	-	-0.06 ± 0.02

Figure 1: The Fen complex with bedrock: colour info. X and Y axis are eastern and northern UTM 32 GPS coordinates and the map has a WGS84 projection.

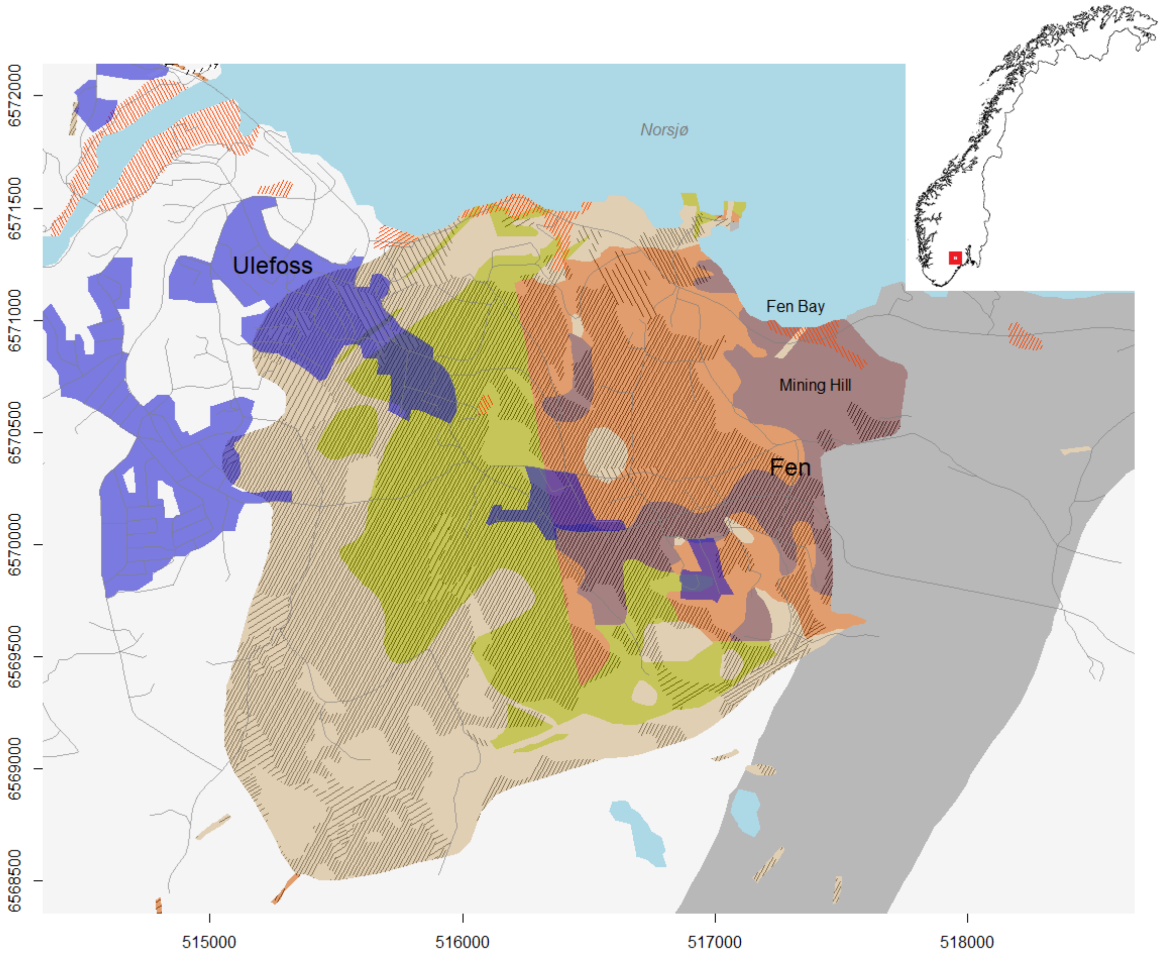

Figure 2 Amount of dried litter filled in fine-meshed and coarser-meshed litterbags before one deployment.

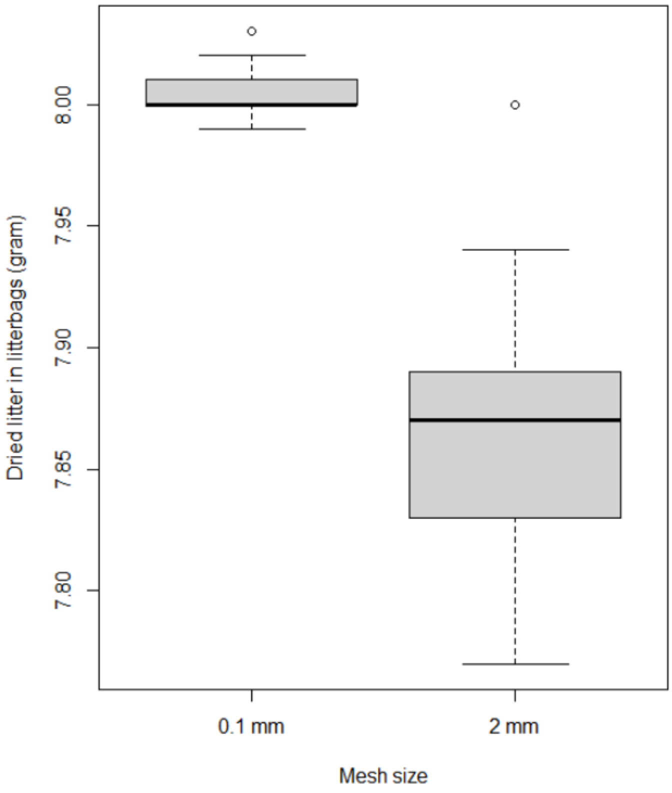

Figure 3 Boxplot of size fraction >2 mm of soil samples among localities within Mining hill.

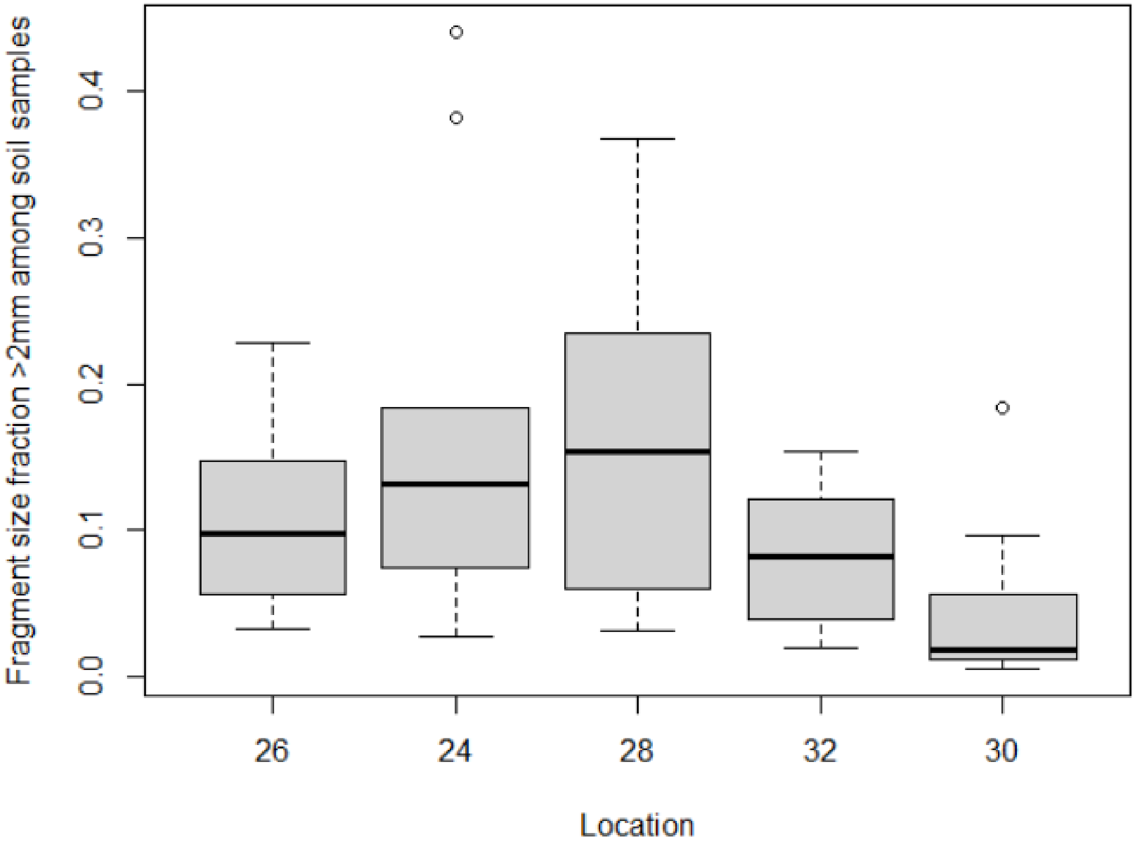

Figure 4 Boxplot of pH of soil samples among localities within Mining hill.

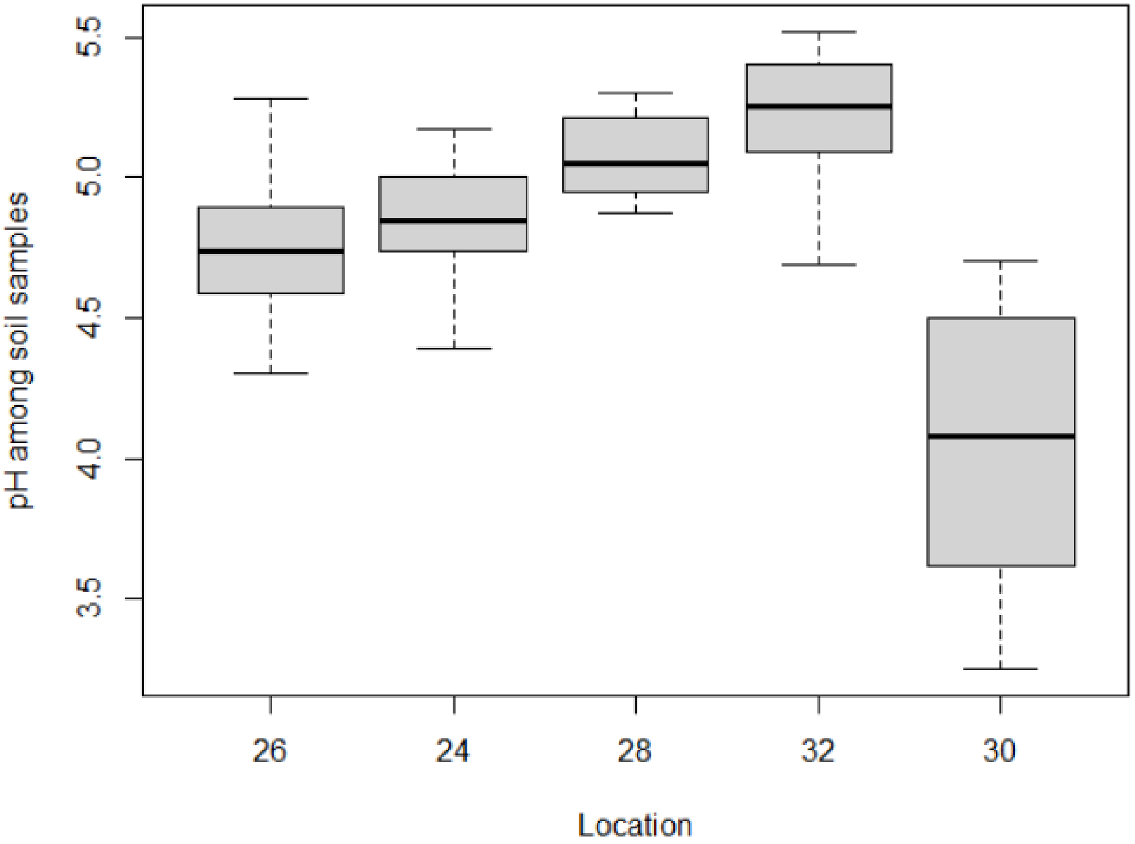

Figure 5 Boxplot of organic matter (OM) fraction of soil samples among localities from Mining hill.

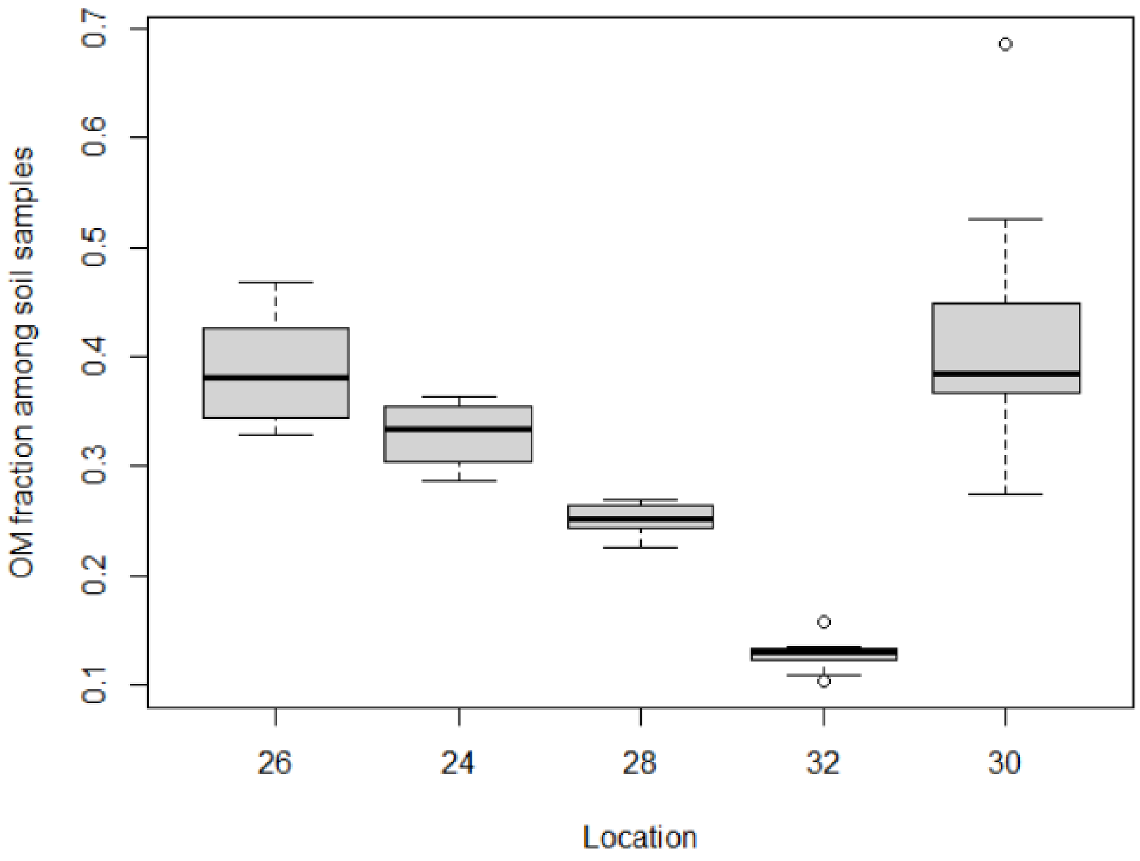

Figure 6 Boxplot of moist fraction of soil samples among localities within Mining hill.

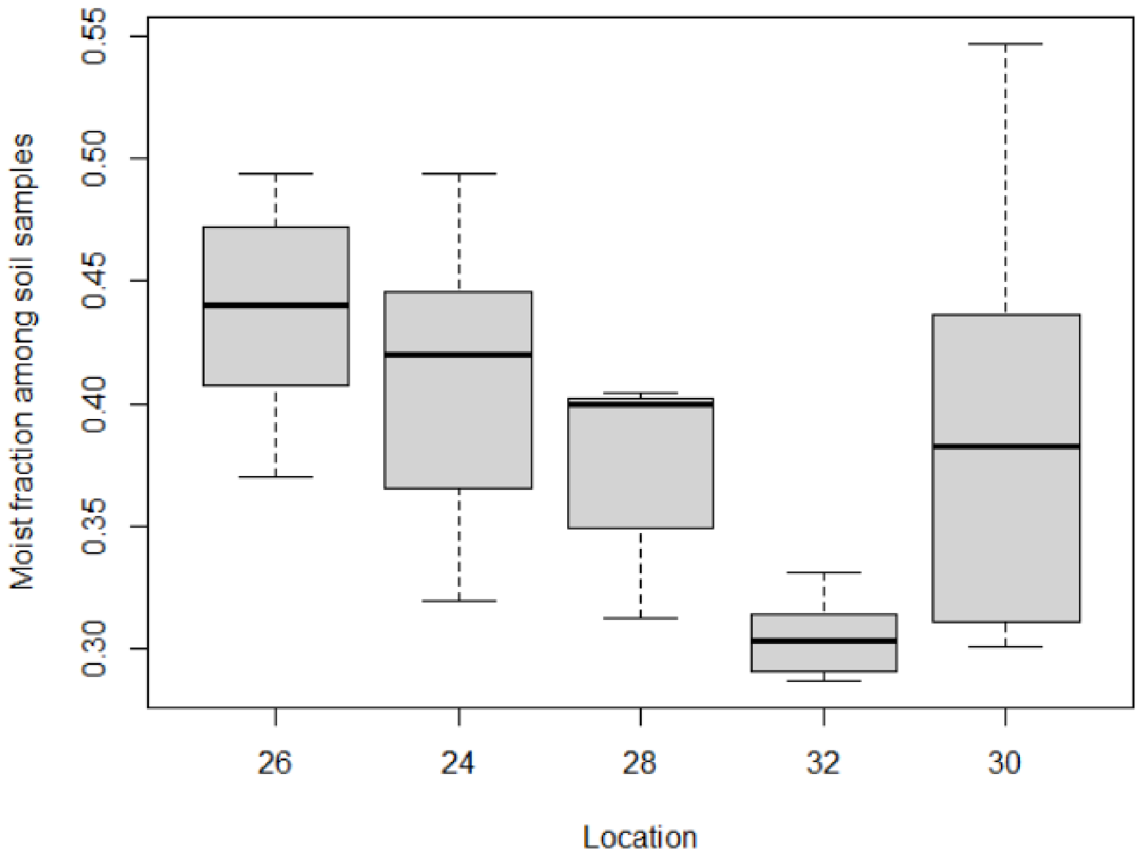

Figure 7 Boxplot of ^{228}Ra (Bq kg^{-1} d.w.) of soil samples among localities within Mining hill.

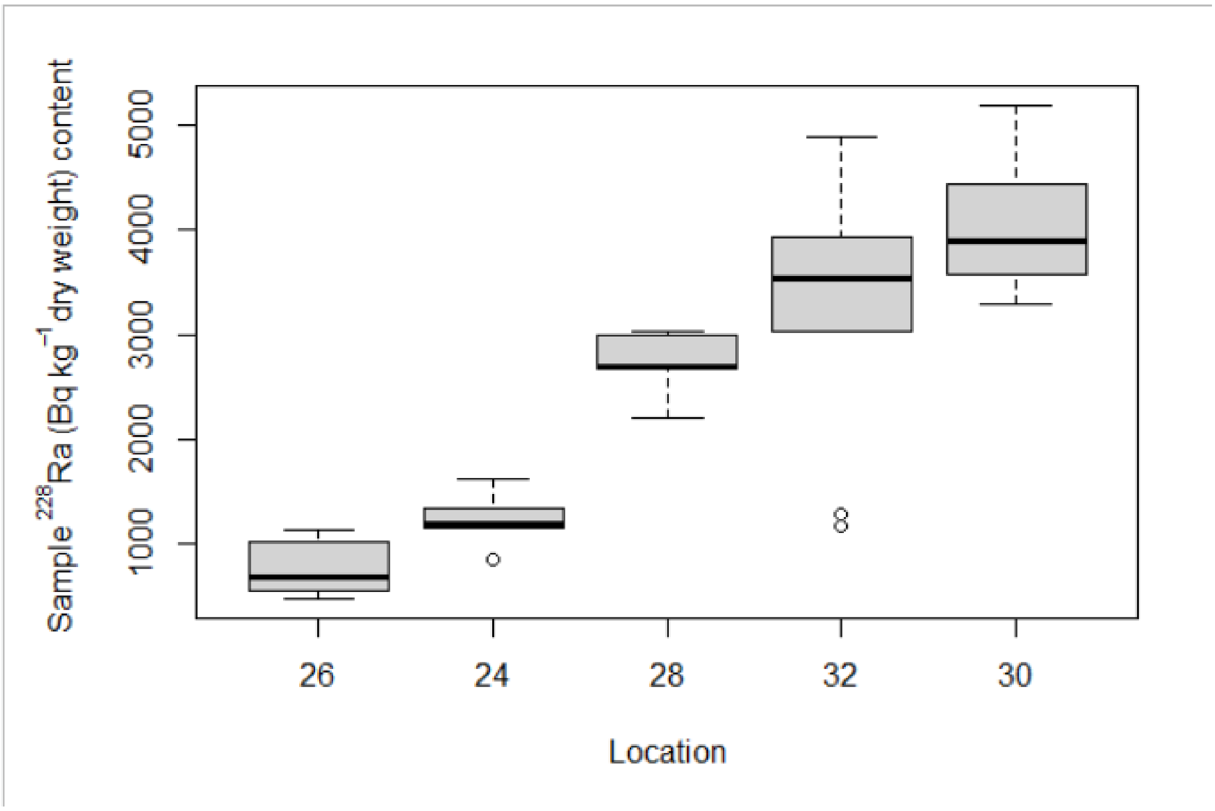

Figure 8 Boxplot of ^{226}Ra (Bq kg^{-1} d.w.) of soil samples among localities within Mining hill.

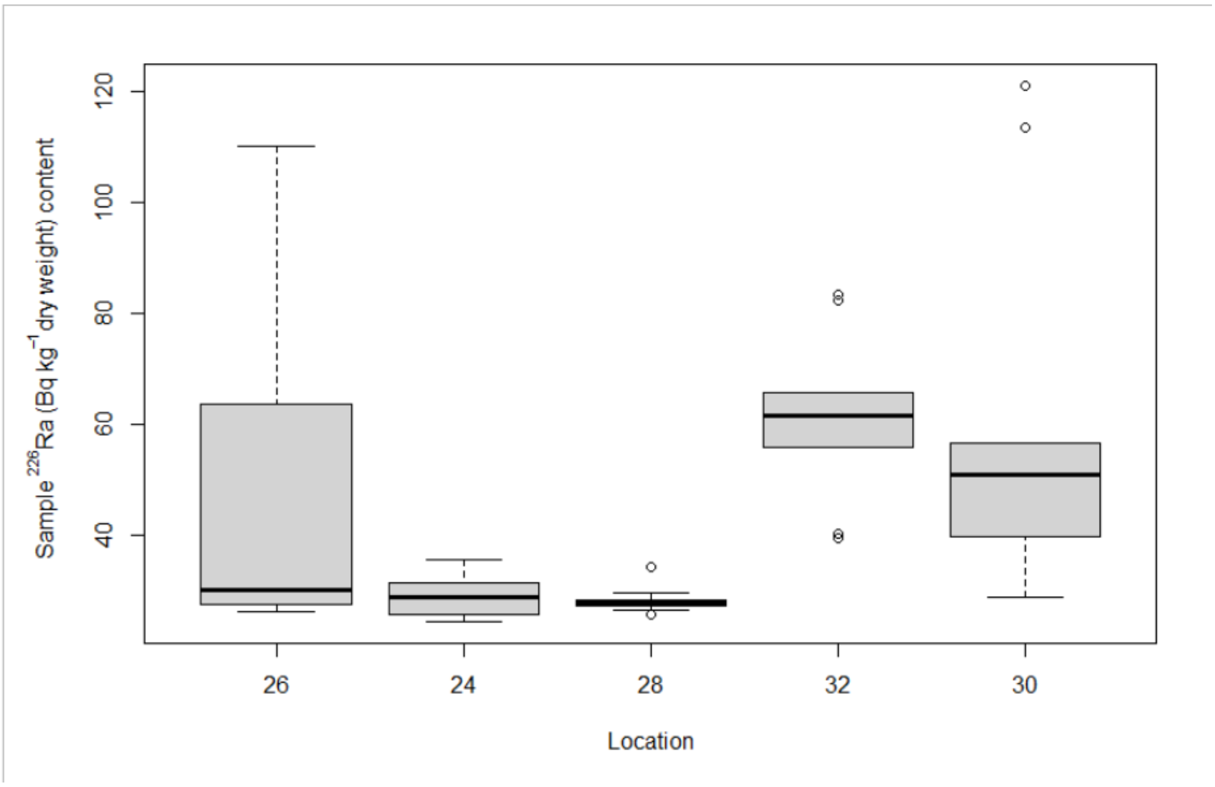

Figure 9 Boxplot of ^{210}Pb to ^{226}Ra ratio of soil samples among localities within Mining hill.

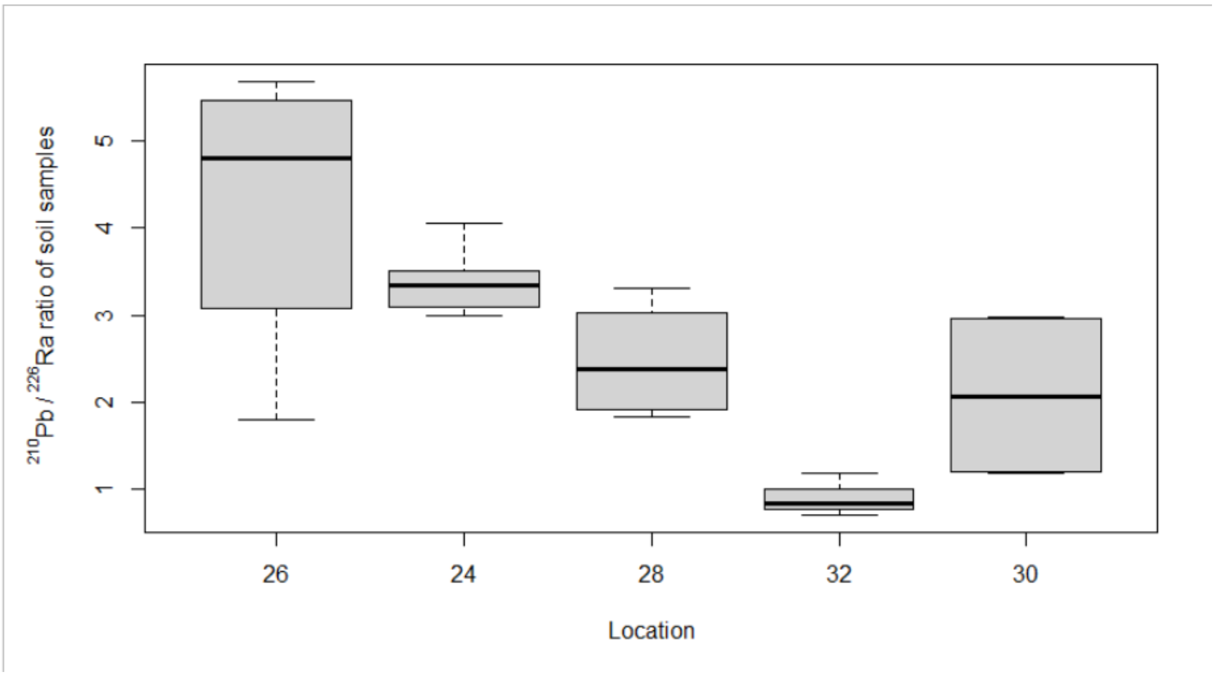

Figure 10 Boxplot of ^{137}Cs (Bq kg^{-1} d.w.) of soil samples among localities within Mining hill.

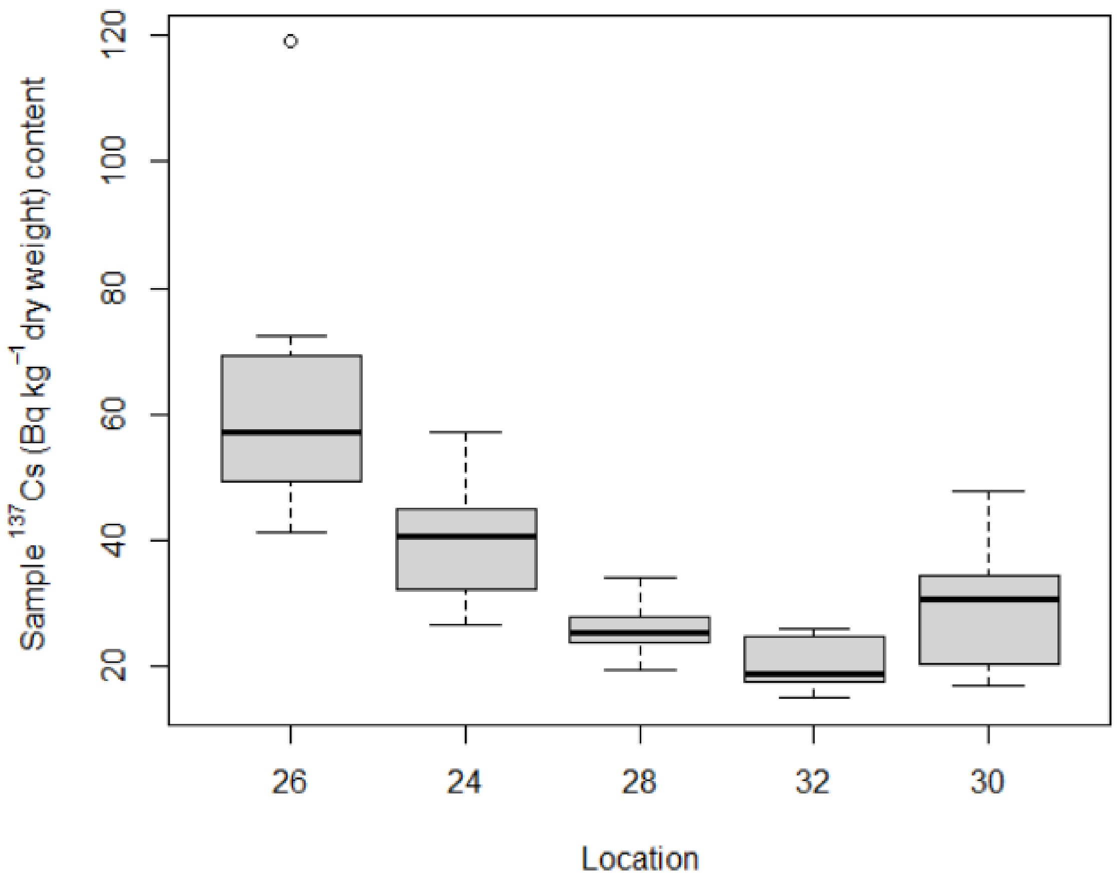

Figure 11 Boxplot of external dose rate estimated with the ERICA tool for an annelid

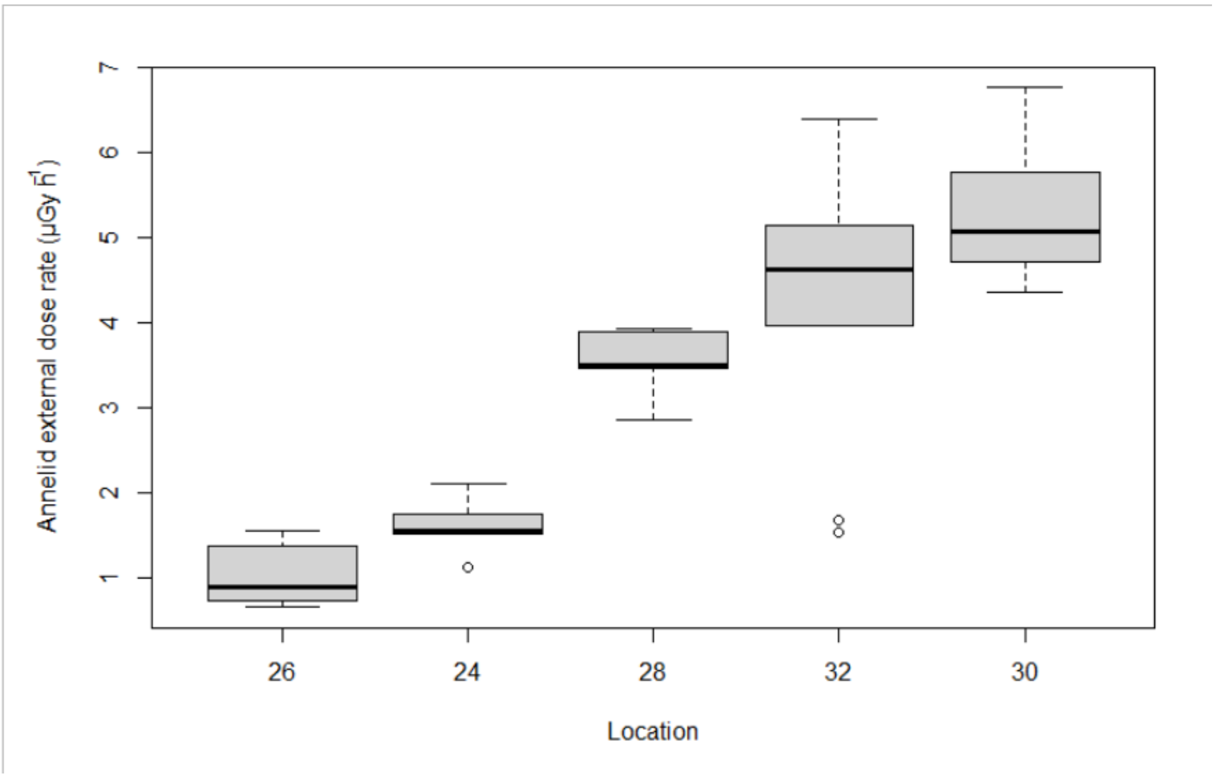

Figure 12 Boxplots of the total dose rate among localities for the four organisms modelled in ERICA tool.

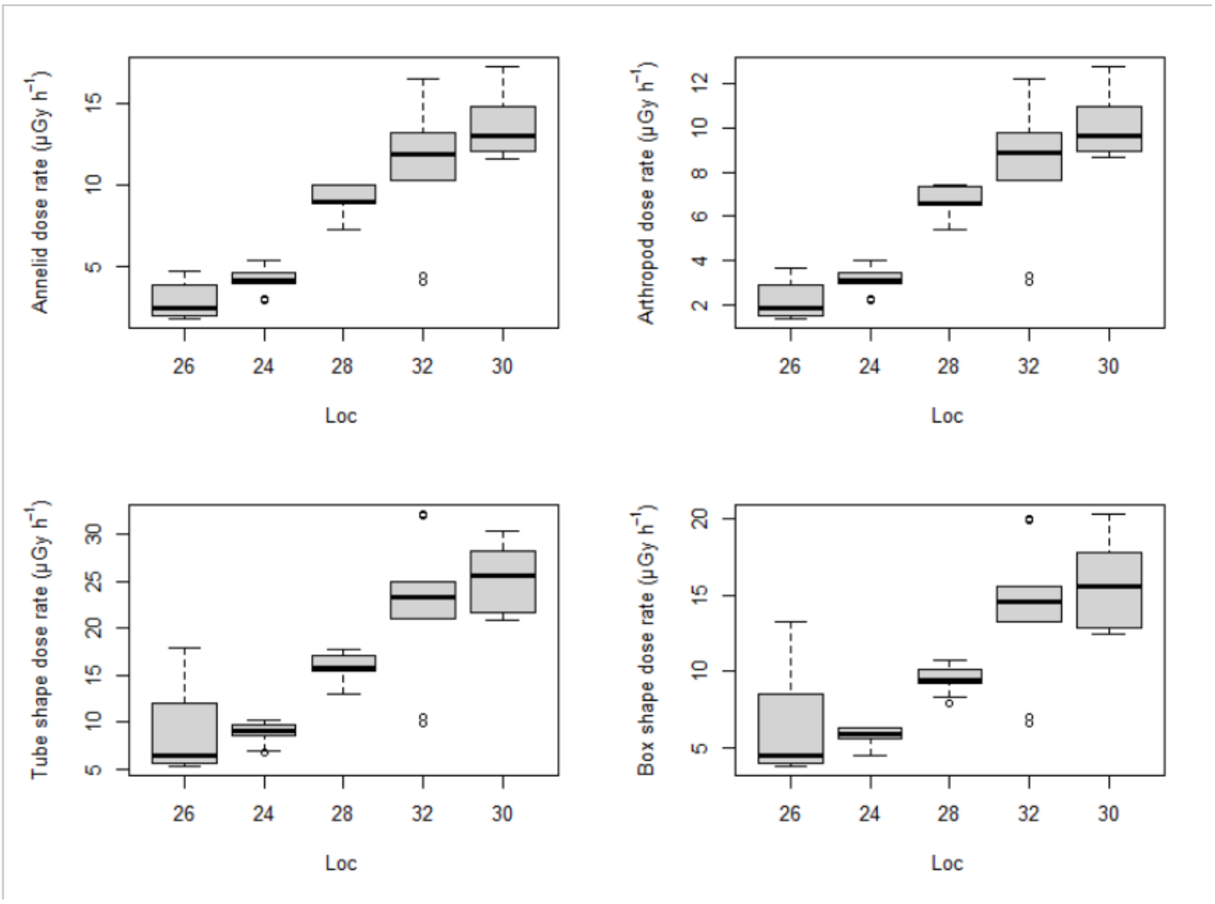

Figure 13 Boxplot of litter loss during one year of exposure from litterbags with coarse (2 mm) and fine (0.1 mm) mesh size when litter was either dried in oven (60° C) or at room temperature (20° C).

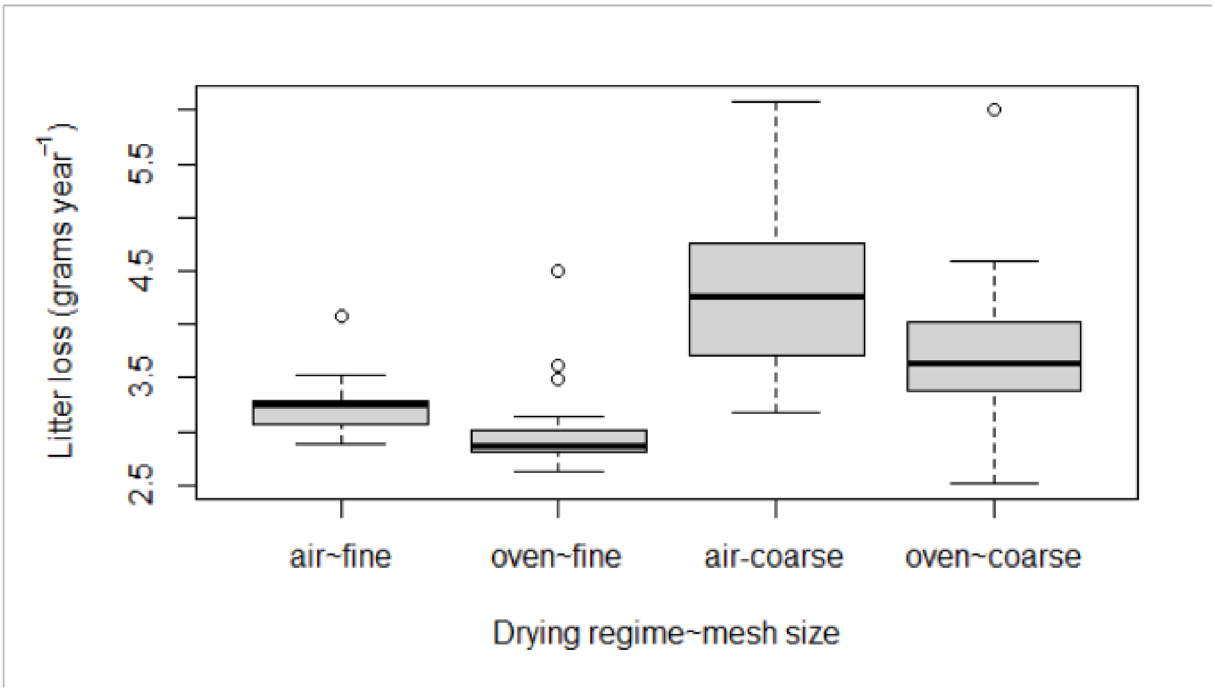

7. Cited literature

1. Lavelle P, Decaëns T, Aubert M, Barot S, Blouin M, et al. (2006) Soil invertebrates and ecosystem services. European Journal of Soil Biology 42, Supplement 1: S3-S15.
2. Barrios E (2007) Soil biota, ecosystem services and land productivity. Ecological Economics 64: 269-285.
3. de Vries FT, Thébault E, Liiri M, Birkhofer K, Tsiafouli MA, et al. (2013) Soil food web properties explain ecosystem services across European land use systems. Proceedings of the National Academy of Sciences 110: 14296-14301.
4. De Deyn GB, Van der Putten WH (2005) Linking aboveground and belowground diversity. Trends in Ecology & Evolution 20: 625-633.
5. Wardle DA, Bardgett RD, Klironomos JN, Setälä H, van der Putten WH, et al. (2004) Ecological Linkages Between Aboveground and Belowground Biota. Science 304: 1629.
6. Bardgett RD (2005) The biology of soil; a community and ecosystem approach. Oxford: Oxford University Press. 242 p.
7. Bardgett RD, Chan KF (1999) Experimental evidence that soil fauna enhance nutrient mineralization and plant nutrient uptake in montane grassland ecosystems. Soil Biology and Biochemistry 31: 1007-1014.
8. Clarholm M (1985) Interactions of bacteria, protozoa and plants leading to mineralization of soil nitrogen. Soil Biology and Biochemistry 17: 181-187.
9. Ingham RE, Trofymow JA, Ingham ER, Coleman DC (1985) Interactions of Bacteria, Fungi, and their Nematode Grazers: Effects on Nutrient Cycling and Plant Growth. Ecological Monographs 55: 119-140.
10. Setälä H (1995) Growth of Birch and Pine Seedlings in Relation to Grazing by Soil Fauna on Ectomycorrhizal Fungi. Ecology 76: 1844-1851.
11. Setälä H, Huhta V (1991) Soil fauna increase *Betula pendula* growth: laboratory experiments with coniferous forest floor. Ecology 72: 665-671.
12. Begon M, Harper JL, Townsend CR (1996) Ecology, individuals, populations and communities. Oxford, UK: Blackwell Science. 1065 p.
13. Haanes H, Hansen EL, Hevrøy TH, Jensen LK, Gjelsvik R, et al. (2019) Realism and usefulness of multispecies experiment designs with regard to application in radioecology: A review. Science of The Total Environment: 134485.
14. Fleeger JW, Carman KR, Nisbet RM (2003) Indirect effects of contaminants in aquatic ecosystems. Science of The Total Environment 317: 207-233.
15. Van den Brink PJ, Tarazona JV, Solomon KR, Knacker T, Van den Brink NW, et al. (2005) The use of terrestrial and aquatic microcosms and mesocosms for the ecological risk assessment of veterinary medical products. Environmental Toxicology and Chemistry 24: 820-829.
16. Folke C, Carpenter S, Walker B, Scheffer M, Elmqvist T, et al. (2004) Regime Shifts, Resilience, and Biodiversity in Ecosystem Management. Annual Review of Ecology, Evolution, and Systematics 35: 557-581.
17. Hooper DU, Chapin FS, Ewel JJ, Hector A, Inchausti P, et al. (2005) EFFECTS OF BIODIVERSITY ON ECOSYSTEM FUNCTIONING: A CONSENSUS OF CURRENT KNOWLEDGE. Ecological Monographs 75: 3-35.
18. Kampichler C, Bruckner A (2009) The role of microarthropods in terrestrial decomposition: a meta-analysis of 40 years of litterbag studies. Biol Rev Camb Philos Soc 84: 375-389.
19. González G, Seastedt TR (2001) Soil Fauna and Plant Litter Decomposition in Tropical and Subalpine Forests. Ecology 82: 955-964.
20. Huhta V (2007) The role of soil fauna in ecosystems: A historical review. Pedobiologia 50: 489-495.

21. Peng Y, Yang W, Li J, Wang B, Zhang C, et al. (2015) Contribution of Soil Fauna to Foliar Litter-Mass Loss in Winter in an Ecotone between Dry Valley and Montane Forest in the Upper Reaches of the Minjiang River. PLoS ONE 10: e0124605.
22. Zhang W, Yuan S, Hu N, Lou Y, Wang S (2015) Predicting soil fauna effect on plant litter decomposition by using boosted regression trees. Soil Biology and Biochemistry 82: 81-86.
23. Frouz J, Roubíčková A, Heděnec P, Tajovský K (2015) Do soil fauna really hasten litter decomposition? A meta-analysis of enclosure studies. European Journal of Soil Biology 68: 18-24.
24. Bokhorst S, Wardle DA (2013) Microclimate within litter bags of different mesh size: Implications for the 'arthropod effect' on litter decomposition. Soil Biology and Biochemistry 58: 147-152.
25. Cárcamo HA, Prescott CE, Chanway CP, Abe TA (2001) Do soil fauna increase rates of litter breakdown and nitrogen release in forests of British Columbia, Canada? Canadian Journal of Forest Research 31: 1195-1204.
26. Swift MJ, Heal OW, Anderson JM (1979) Decomposition in terrestrial ecosystems. Oxford: Blackwell.
27. Briones MJI (2014) Soil fauna and soil functions: a jigsaw puzzle. Frontiers in Environmental Science 2.
28. Wang S, Ruan H, Han Y (2010) Effects of microclimate, litter type, and mesh size on leaf litter decomposition along an elevation gradient in the Wuyi Mountains, China. Ecological Research 25: 1113-1120.
29. Förster B, Garcia M, Francimari O, Römbke J (2006) Effects of carbendazim and lambda-cyhalothrin on soil invertebrates and leaf litter decomposition in semi-field and field tests under tropical conditions (Amazônia, Brazil). European Journal of Soil Biology 42, Supplement 1: S171-S179.
30. Parmelee RW, Wentsel RS, Phillips CT, Checkai RT, Simini M (1993) Soil microcosm for testing the effects of chemical pollutants on soil fauna communities and trophic structure. Environmental Toxicology and Chemistry 12: 1477-1486.
31. Burrows LA, Edwards CA (2002) The use of integrated soil microcosms to predict effects of pesticides on soil ecosystems. European Journal of Soil Biology 38: 245-249.
32. Edwards CA, Subler S, Parmelee R, Knacker T, Pokarzhevskij AA (1997) Use of soil microcosms in assessing the effect of pesticides on soil ecosystems. International Atomic Energy Agency (IAEA): IAEA.
33. Mousseau TA, Milinevsky G, Kenney-Hunt J, Moller AP (2014) Highly reduced mass loss rates and increased litter layer in radioactively contaminated areas. Oecologia 175: 429-437.
34. Bonzom J-M, Hättenschwiler S, Lecomte-Pradines C, Chauvet E, Gaschak S, et al. (2016) Effects of radionuclide contamination on leaf litter decomposition in the Chernobyl exclusion zone. Science of The Total Environment 562: 596-603.
35. Zaitsev AS, Gongalsky KB, Nakamori T, Kaneko N (2014) Ionizing radiation effects on soil biota: Application of lessons learned from Chernobyl accident for radioecological monitoring. Pedobiologia 57: 5-14.
36. Setälä H, Huhta V (1990) Evaluation of the soil fauna impact on decomposition in a simulated coniferous forest soil. Biology and Fertility of Soils 10: 163-169.
37. Andre A, Antunes SC, Goncalves F, Pereira R (2009) Bait-lamina assay as a tool to assess the effects of metal contamination in the feeding activity of soil invertebrates within a uranium mine area. Environ Pollut 157: 2368-2377.
38. Freitas AC, Rodrigues D, Rocha-Santos TAP, Gonçalves F, Duarte AC, et al. (2014) The Impact of Uranium Mine Contamination of Soils on Plant Litter Decomposition. Archives of Environmental Contamination and Toxicology 67: 601-616.

39. Popic JM, Bhatt CR, Salbu B, Skipperud L (2012) Outdoor 220Rn, 222Rn and terrestrial gamma radiation levels: investigation study in the thorium rich Fen Complex, Norway. Journal of environmental monitoring 14: 193-201.
40. Stranden E (1985) The radiological impact of mining in a Th-rich Norwegian area. Health physics 48: 415-420.
41. Popic JM, Meland S, Salbu B, Skipperud L (2014) Mobility of radionuclides and trace elements in soil from legacy NORM and undisturbed naturally 232Th-rich sites. Environ Sci Process Impacts 16: 1124-1134.
42. Popic JM, Salbu B, Strand T, Skipperud L (2011) Assessment of radionuclide and metal contamination in a thorium rich area in Norway. J Environ Monitor 13: 1730-1738.
43. Cagno S, Lind OC, Popic JM, Skipperud L, De Nolf W, et al. (2020) Micro-analytical characterization of thorium-rich aggregates from Norwegian NORM sites (Fen Complex, Telemark). Journal of Environmental Radioactivity 219: 106273.
44. Popic JM, Salbu B, Skipperud L (2012) Ecological transfer of radionuclides and metals to free-living earthworm species in natural habitats rich in NORM. Sci Total Environ 414: 167-176.
45. Haanes H, Rudjord AL (2018) Significance of seasonal outdoor releases of thoron from airflow through a point source during natural ventilation of a mine-complex in thorium-rich bedrock. Atmospheric Pollution Research 9: 1000-1008.
46. Moody SA, Paul ND, Björn LO, Callaghan TV, Lee JA, et al. (2001) The direct effects of UV-B radiation on Betula pubescens litter decomposing at four European field sites. Plant Ecology 154: 27-36.
47. Pathak JP, Macmillan RH, Evans DG, Murray DR (1978) The effects of temperature on the retention and denaturation of protein in lucerne leaves subjected to mechanical dewatering. Journal of the Science of Food and Agriculture 29: 835-838.
48. Brown JE, Alfonso B, Avila R, Beresford NA, Copplestone D, et al. (2016) A new version of the ERICA tool to facilitate impact assessments of radioactivity on wild plants and animals. Journal of Environmental Radioactivity 153: 141-148.
49. Lourenço J, Pereira R, Silva A, Carvalho F, Oliveira J, et al. (2012) Evaluation of the sensitivity of genotoxicity and cytotoxicity endpoints in earthworms exposed in situ to uranium mining wastes. Ecotoxicology and environmental safety 75: 46-54.
50. Ohtsuka Y, Takaku Y, Hisamatsu Si (2015) Background internal dose rates of earthworm and arthropod species in the forests of Aomori, Japan. Journal of Radioanalytical and Nuclear Chemistry 303: 1441-1445.
51. Pokarzhevskii AD, Krivolutzkii DA (1997) Background concentration of 226Ra in terrestrial animals. Biogeochemistry 39: 1-13.
52. Törmälä T (1979) Numbers and biomass of soil invertebrates in a reserved field in central Finland. Agricultural and Food Science 51.
53. Bezuidenhout J (2020) The investigation of natural radionuclides as tracers for monitoring sediment processes. Journal of Applied Geophysics 181: 104135.
54. Gale HJ (1967) Some Examples of the Application of the Lognormal Distribution in Radiation Protection. The Annals of Occupational Hygiene 10: 39-45.
55. Haanes H, Finne IE, Kolstad T, Mauring A, Dahlgren S, et al. (2016) Outdoor thoron and progeny in a thorium rich area with old decommissioned mines and waste rock. Journal of Environmental Radioactivity 162–163: 23-32.
56. Haanes H, Finne IE, Skjerdal HK, Rudjord AL (2019) Indoor and Outdoor Exposure to Radon, Thoron and Thoron Decay Products in a NORM Area with Highly Elevated Bedrock Thorium and Legacy Mines. Radiation Research 192: 431-439.
57. R Core Team (2016) R: A language and environment for statistical computing. In: Computing RFfS, editor. Vienna, Austria. URL https://www.R-project.org/.

58. Burnham KP, Anderson DR (2002) Model selection and multimodel inference. A practical information-theoretic approach, 2nd edn. Berlin Heidelberg New York: Springer.
59. Hättenschwiler S, Gasser P (2005) Soil animals alter plant litter diversity effects on decomposition. Proceedings of the National Academy of Sciences of the United States of America 102: 1519-1524.
60. Nielsen GA, Hole FD (1964) Earthworms and the Development of Coprogenous A1 Horizons in Forest Soils of Wisconsin. Soil Science Society of America Journal 28: 426-430.
61. Aber JD, Melillo JM, McClaugherty CA (1990) Predicting long-term patterns of mass loss, nitrogen dynamics, and soil organic matter formation from initial fine litter chemistry in temperate forest ecosystems. Canadian Journal of Botany 68: 2201-2208.
62. Couˆteaux M-M, Bottner P, Berg B (1995) Litter decomposition, climate and liter quality. Trends in Ecology & Evolution 10: 63-66.
63. Gholz HL, Wedin DA, Smitherman SM, Harmon ME, Parton WJ (2000) Long-term dynamics of pine and hardwood litter in contrasting environments: toward a global model of decomposition. Global Change Biology 6: 751-765.
64. Lavelle P, Chauvel A, Fragoso C (1995) Faunal activity in acid soils. In: Date RA, Grundon NJ, Rayment GE, Probert ME, editors. Plant-Soil Interactions at Low pH: Principles and Management: Proceedings of the Third International Symposium on Plant-Soil Interactions at Low pH, Brisbane, Queensland, Australia, 12–16 September 1993. Dordrecht: Springer Netherlands. pp. 201-211.
65. Keith LS, Faroon OM, Fowler BA (2007) CHAPTER 45 - Uranium. In: Nordberg GF, Fowler BA, Nordberg M, Friberg LT, editors. Handbook on the Toxicology of Metals (Third Edition). Burlington: Academic Press. pp. 881-903.
66. Keith S, Faroon O, Roney N, Scinicariello F, Wilbur S, et al. (2013) Toxicological Profile for Uranium. Atlanta GA: U.S. DEPARTMENT OF HEALTH AND HUMAN SERVICES, Agency for Toxic Substances and Disease Registry.
67. Vandenhove H, Gil-García C, Rigol A, Vidal M (2009) New best estimates for radionuclide solid–liquid distribution coefficients in soils. Part 2. Naturally occurring radionuclides. Journal of Environmental Radioactivity 100: 697-703.
68. Simon SL, Ibrahim SA (1987) The plant/soil concentration ratio for calcium, radium, lead, and polonium: Evidence for non-linearity with reference to substrate concentration. Journal of Environmental Radioactivity 5: 123-142.
69. IAEA (2014) The Environmental Behaviour of Radium: Revised Edition. Vienna: INTERNATIONAL ATOMIC ENERGY AGENCY.
70. Koch-Steindl H, Pröhl G (2001) Considerations on the behavior of long-lived radionuclides in the soil. Radiat Environ Biophys 40: 93-104.
71. Antunes SC, Pereira R, Marques SM, Castro BB, Goncalves F (2011) Impaired microbial activity caused by metal pollution: A field study in a deactivated uranium mining area. Sci Total Environ 410-411: 87-95.
72. Dorsey AF, Fransen ME, Diamond GL, Amata RJ (2004) Toxicological profile of strontium. U.S. DEPARTMENT OF HEALTH AND HUMAN SERVICES, Public Health Service Agency for Toxic Substances and Disease Registry.
73. Lai J-l, Tao Z-y, Fu Q, Han N, Wu G, et al. (2016) The chemical toxicity of cesium in Indian mustard (Brassica juncea L.) seedlings. Journal of Environmental Radioactivity 160: 93-101.
74. Williams M, Wohlers DW, Citra M, Diamond GL, Swarts SG (2004) Toxicological profile of cesium. U.S. DEPARTMENT OF HEALTH AND HUMAN SERVICES, Public Health Service Agency for Toxic Substances and Disease Registry.
75. Cragg RG, Bardgett RD (2001) How changes in soil faunal diversity and composition within a trophic group influence decomposition processes. Soil Biology and Biochemistry 33: 2073-2081.

76. Hättenschwiler S, Tiunov AV, Scheu S (2005) Biodiversity and litter decomposition in terrestrial ecosystems. Annual Review of Ecology, Evolution, and Systematics 36: 191-218.
77. Brechignac F, Bradshaw C, Carroll S, Fuma S, Håkanson L, et al. (2012) Towards an Ecosystem Approach for Environmental Protection with Emphasis on Radiological Hazards.: IUR; International Union of Radioecology.
