# Supplementary material for: Reduced soil fauna decomposition in a high background radiation area
Source: PLoS One. 2021 Mar 17;16(3):e0247793. doi: 10.1371/journal.pone.0247793 (PMC7968631; doi:10.1371/journal.pone.0247793)
Supplement: Attachment — Submitted filename: Rebuttal letter5feb.docx [file pone.0247793.s003.docx]

Rebuttal letter

- *with editor and reviewers comments in italic* and our responses brown in plain text

***EDITOR****s comments*

*Thank you for submitting your manuscript to PLOS ONE. After careful consideration, we feel that it has merit but does not fully meet PLOS ONE’s publication criteria as it currently stands. Therefore, we invite you to submit a revised version of the manuscript that addresses the points raised during the review process.*

*This a wonderful experiment that attempts to get at several different issues related to decomposition and radiation in a fundamentally important ecological process, decomposition. The authors have put a lot of effort into their design and analyses and these findings are thus well worth reporting. There were however a few issues that will need to be addressed before the paper will be ready for publication. It is not clear to me if all of these issues can be addressed but the authors should do their best!*

We thank the editor for the positive feedback, for the comments to improve the manuscript and the possibility to revise and resubmit our manuscript. We have accommodated to all comments to our best ability. All changes are shown in one version with track changes, but we also include a clean version.

*From my perspective, there were a few important aspects of the study design that were not reported. First, although the idea of testing for differences in heat treatment (and thus extent of protein denaturalization) is a worthy question, it was not clear that this was addressed sufficiently. Was extent of denaturization measured?*

We really appreciate the need to include sufficient information about all aspects of study design, both on applied conditions and for assumptions and associated theoretical aspects. As such, the effect of temperature treatment on leaf protein denaturization is relatively well known. As an example, both Phatak et al (1978) and Fu et al (2010) show how the extent of protein denaturization in leaves increases with temperature as temperature increases from around 30⁰C to 80⁰C, at which point approximately all protein has denaturized and become less accessible to extraction (see figure 3 in Phatak et al 1978 and figure 4 in Fu et al 2010). It should be safe to assume a similar relationship for the denaturization of proteins in birch leaves with increasing temperature and that an equal amount/extent of protein denaturization will occur in all leaves treated with the same temperature (60 ⁰C) for equal periods of time. To accommodate to this comment, we have therefore added this information on extent of denaturization with temperature, citing these two references, to clarify. We have also included in our discussion that decreased availability of leaf proteins as an alternative explanation to lower decomposition in both coarse and fine meshed litterbags (see also below).

*Did the authors compare water content of leaf material from both oven and air dried samples? Air dried samples are unlikely to get any drier than ambient (unless placed in containers with drierite desiccant) while oven dried samples can get quite dry! Could this perhaps account for some of the findings?*

We did assess the amount of water content in both fresh litter and in litter dried in room temperature (air-dried) and in litter dried in oven To accommodate, we have now included these numbers in the methods section to show that air-dried litter contained closely as little water as oven-dried litter. This was probably due to us using an old and very dry storage facility for drying at room-temperature.

*I would like to see data on % loss reported as well. It looked like some of the bags (large mesh only) lost 100% of their material. Is this correct?*

To accommodate, in addition to report on the actual mass of litter loss, we have now included results on the percent of litter loss from litterbags, including the range (minimum and maximum values) median, mean and standard deviation for the fraction of dried litter lost from coarse-meshed and fine-meshed litterbags. As you can see from the results, maximum loss was 78% and any litterbags that lost over 56% had a coarse mesh.

*Related to this, what did the authors do to minimize the loss of small particle sizes through the larger mesh holes during transport? What was done to minimize the loss due to mechanical factors over the course of the year as opposed to true decomposition effects? Were invertebrates counted in both bag types to demonstrate that this was indeed the cause of the differences?*

After filling the litterbags, these were all kept in four large bags (IKEA type) until each litterbag was placed on the soil in Fen. These large bags are very sturdy and have no holes. To assess whether any fragments of litter were lost through the wholes of the coarse meshed litterbags during transport, we carefully inspected the bottom of these bags after the litterbags had been deployed. The amount of fragments detected was close to zero and negligible. The same was done and observed when the litterbags were recollected and transported back to the laboratory. Furthermore, from each litterbag field position, litterbags were transported in separate aluminium trays. We are sorry that we did not mention this in our original manuscript and to accommodate, we have included information on this in the revision. When we re-collected the litterbags, there were no signs that any of them had been moved, rather debris etc on the litterbags indicated they had lied perfectly still during deployment. We have now included information also about this.

Moreover, working with our response to the editor comment on extent of protein denaturization. made us think about how denaturization making proteins less available to extraction also would make the proteins less available to digestion in animals. This provides an additional or alternative explanation to the slower rate of litter loss from oven-dried litter compared to room-dried litter, which in the submitted manuscript was suggested to be due to the increased stiffness of oven dried litter that may reduce shredding and eating efficiencies of soil fauna. We realized that this also gives good information to whether litter fragments may be lost from coarsely meshed bags due to fragmentation from physical and mechanical wear. The more brittle oven-dried litter should then have involved more fragmentation and loss than the room-temperature litter, but the opposite was the case (see results). Also, the difference in decomposition rate between room-temperature dried and oven-dried litter was also found among the fine meshed litterbags, which we now have included. This indicates that the brittleness and/or denaturization of heating litter also affects microbe decomposition, which we therefore have included a comment on in the discussion section.

We counted invertebrates in litterbags that were large enough to be spotted by eye. However, the fine meshed litterbags (0.1 mm) were sealed with glue and have a so fine mesh size that nothing larger than 0.1 mm can enter. We observed no mesofauna in the fine meshed litterbags. To accommodate, we now mention this in the methods and result sections, as well as the worms we found in four of the coarse meshed litterbags (results section).

*The authors mention Chernobyl as a context for their work but I don't believe any comparisons are generated between radiation levels in Norway vs Chernobyl. My guess is that the differences are rather large…. How might this be reflected in the findings? Is the range of radiation levels used in this experiment great enough to test for radiation effects given the constraints on sample size?*

To accommodate, we now in the discussion mention the levels of anthropogenic radioactivity in the two Chernobyl litterbag studies and compare these to the levels in our study. As the editor suggests, these were higher than in our study, but they were from anthropogenic radionuclides rather than natural radionuclides. One aim of our study was to assess whether the range of naturally occurring radionuclides in Fen is great enough to involve effects on soil fauna decomposition.

Our study does have fewer parallels (litterbags) than the two Chernobyl studies, but we do have a very nice resolution in our predictor variables. This is because soil samples were taken beneath each of the 50 coarse meshed litterbags, so that variation in litter decomposition within these bags can be related to variation in each of these 50 soil sample parameters. This is quite relative given the very high degree of spatial heterogeneity of natural radionuclides at Fen, where levels can vary quite a lot between litterbags in just different quartets (within the same locality, see table 2). By comparison, Cs deposition from air involves far less spatial heterogeneity and the two Chernobyl studies thus used pooled measures of radioactivity and soil parameters for their 20 and 11 study sites, respectively. We hope the editor appreciate that our experimental design thus is very well designed to detect covariation between soil parameters and litter decomposition and that there was no constraint on sample size. To clear up we have included an additional sentence in this discussion chapter about how this high spatial heterogeneity allows us to compare the variation in soil parameters (including radionuclides) among 50 coarse meshed litterbags with their variation in litter decomposition (see also our comment to one of the comments by reviewer2 on comparison of all litterbags).

*The two reviewers also have a few suggestions for improvement that should be addressed in your revision.*

The two reviewers have very different perspectives, but we have tried to accommodate both reviewer’s comments as far as possible and to our best ability.

*Please submit your revised manuscript by Mar 05 2021 11:59PM. If you will need more time than this to complete your revisions, please reply to this message or contact the journal office at* [*plosone@plos.org*](mailto:plosone@plos.org)*. When you're ready to submit your revision, log on to* [*https://www.editorialmanager.com/pone/*](https://www.editorialmanager.com/pone/) *and select the 'Submissions Needing Revision' folder to locate your manuscript file.
Please include the following items when submitting your revised manuscript:*

- *A rebuttal letter that responds to each point raised by the academic editor and reviewer(s). You should upload this letter as a separate file labeled 'Response to Reviewers'.*
- *A marked-up copy of your manuscript that highlights changes made to the original version. You should upload this as a separate file labeled 'Revised Manuscript with Track Changes'.*
- *An unmarked version of your revised paper without tracked changes. You should upload this as a separate file labeled 'Manuscript'.*

We have now prepared a rebuttal letter (this document), as well as a revision with track changes (Revision_track_changes.doc) and a clean revision (Revision_clean.doc). See also the cover letter.

*If you would like to make changes to your financial disclosure, please include your updated statement in your cover letter. Guidelines for resubmitting your figure files are available below the reviewer comments at the end of this letter.*

The funding statement should read as it did in the original acknowledgement section (below).

"This work was supported by the Research Council of Norway through its Centres of Excellence
465 funding scheme, project number 223268/F50. This was received by HH and RG at Norwegian Radiation and Nuclear Safety Authority (DSA) thorough the Centre for
466 Environmental Radioactivity, CERAD, Centre of Excellence CoE (<https://www.nmbu.no/en/services/centers/cerad)>." We have also followed figure guidelines (see our responses at the end of the letter).

*If applicable, we recommend that you deposit your laboratory protocols in protocols.io to enhance the reproducibility of your results. Protocols.io assigns your protocol its own identifier (DOI) so that it can be cited independently in the future. For instructions see:* [*http://journals.plos.org/plosone/s/submission-guidelines#loc-laboratory-protocols*](http://journals.plos.org/plosone/s/submission-guidelines#loc-laboratory-protocols)

*REVIEWER1*

*Reviewer #1: The manuscript "Reduced soil fauna decomposition in a high background radiation area" by Hallvard Haanes and Runhild Gjelsvik, deals with the study of the radioactivity impact on soil fauna using the litter decomposition test. The topic is important due to needs to understand how the high radioactive contamination in areas of Chornobyl and Fukushima disasters as well as in areas of the natural enhanced radioactivity effects on ecosystem.

General comment

In general the manuscript is interesting, useful and good written; provides informative and good investigation. However, the manuscript should be slightly corrected. The sentences (Line 24) that "results on effects of anthropogenic radionuclides on soil fauna decomposition in Chernobyl are contradictory" and (L83-84) "litterbags have been used to both verify deleterious effects of anthropogenic radioactive pollution [33], as well as contradict them [34]" should be updated and clarified. Actually, if compare the statistics and range of study (by radioactivity level, spatial coverage) of two experiments in [33] and [34], rather results of [34] is doubtful. So I would propose authors to look at these papers more deep and update some details in these sentences.
The Chornobyl disaster radioactive contamination extended on Ukraine, Belarus and Russia areas. Therefore need to be more precise indicating the study area (see L85 "from Russian spill sites").*

We thank reviewer1 for kind feedback and helpful comments to improve our manuscript. We acknowledge and apologise that our statements in lines 24 and lines 83-84 didn’t truly reflect the content of the cited references. Having compared in detail their field experiment conditions, methodology, measurements techniques and radiation levels, we absolutely agree with reviewer 1. We have therefore rephrased these sentences in the abstract and the introduction and now do not here focus on the different results of these two references. Rather we focus on the few assessments there have been on effects of radioactivity on soil fauna from radioactivity and that these have been for anthropogenic radioactivity.

To accommodate to this comment by reviewer1 on the differences between these references, we mention this in the discussion (L: ) where Chernobyl results were discussed in the originally submitted manuscript (see also our responses to one comment by the editor and two comments by reviewer2). Now, we focus on how the different results of these references probably are due to their different methodology with differences in level of radioactivity, spatial coverage, litter species, drying regime, litter bag mesh size, soil parameters and statistical analyses. We do not want to judge the results of other studies, so we only point out how some of these differences may have large effects on results. For example, different levels and ranges of radioactivity could be expected to affect their results, the very large mesh size of the litterbags in reference 34 allows also macrofauna into their litterbags, and the study at hand demonstrates that the different litter drying temperatures may affect decomposition.

We have also replaced “Russian” with Soviet according to other minor comments by both reviewer1 and reviewer2.

*Minor comments
in Editorial Page 1
Abstract: (60 0 C) should be 60C*

This typo is found in the pdf built during submission and is not part of the submitted manuscript text. It is due to the pasted abstract during submission, resulting in a change of format. The text of the submitted manuscript does not have this typo. We will make sure to examine the final version to avoid any format errors occurring during (re-)submission.

*Authors: ‘Haanes Gjelsvik’ should be Runhild Gjelsvik (see Manuscript: P1, L3)*

This typo is found in the pdf built during submission and is not part of the submitted manuscript text. This typo in the name of Runhild Gjelsvik was due to something that went wrong during input of data during submission. We will ensure that our names are correctly spelled in the final version.

*in the manuscript
P3, L54 – Soil fauna affect should be Soil fauna affects*

We have now corrected this typo.

*L70, 119, 122 – N:C or C:N – ??*

We have now corrected this typo.

*P4, L76 – It has been show should be It has been shown*

We have now corrected this typo.

*P4, L85 – Russian (??) – in Abstract [35]: several Soviet era nuclear disasters and the resulting severe radioactive pollution…
… over about 25,000 km2 of the territory of the former USSR alone. (see in General comment above)*

We apologise for the error. The cited author refers to spill sites across the old Soviet. We have now replaced “Russian” with “Soviet Union” according to this comment by reviewer1.

*P5, L100 – (580 mill y) – in SI, (580 Ma) (i.e. Mega annum) is preferable.*

We have now changed this accordingly.

*P5, L124 – September 28 – year have to be mentioned?*

The litter was sampled the same year the litterbags were deployed. We have now included this information.

*P7, L147 – The pattern of litterbags across localities was situated*

We thank for help identifying this typo and have now corrected it.

*P7, L150–151 but elsewise placements within each quartet were random*

We thank for help identifying this typo and have now corrected it.

*P7, L156 – October 17, – year?*

We have now included the year of deployment and collection (2016 to 2017).

*P8, L187 – using 5 hours to reach (what??) and 12 hours at 550C – have to be corrected*

We apologise for this unclear sentence, and we have now clarified to inform that it took 5 hours to reach 550C and that samples were kept at ignition at this temperature for 12h.

*P9, L191 – Cs137 – should be 137(Superscript)Cs*
We thank for help identifying this typo and have now corrected it.

*P9, L206–207 – while Enchytraeids range from 0.5 to 1.3 mm in width and 1 to 40 mm in length and earthworms range from 2 to 20 mm in with and 12 to 80 mm […].
should be: ‘in width and 12 to 80 mm in length […]’.*

We have now corrected the typos in earthworm width and length.

*P9, L208 – ‘the concentration (CR) ratio equals’ should be ‘ the concentration ratio (CR) equals’*

Have been corrected.

*P10, L225 – ‘However, the for the tube-shape’*

We have now corrected this typo and deleted the erroneous “the”.

*P11, L241 – log-transformed should be log(Subscript)10 transformed (subscript as in L257–260) or log(Subscript)10-transformed*

We have now corrected and rephrased this sentence to accommodate. We have also gone through the text replacing and consequently using a hyphen for log_10_-transformed.

*P11, L247–250:
To assess spatial autocorrelation, correlations were made between a) an array of all possible pairwise physical distances all soil samples (n=1225) and b) each of arrays of the corresponding pairwise absolute differences and each of these soil sample pairs for each of the soil parameters. (check this sentence and clarify)*

To assess spatial autocorrelation, we assessed how well differences between soil cores in soil parameters correlated with their corresponding distance (in the pair). We agree the sentence was unclear and we have rephrased accordingly.

*P16, L357–358 – Presence of macrofaunal like earthworms has a substantial effect on…*

We have now corrected this typo.

*P18, L403 – (5.6) and L404 – 4.5e9. – units?*

We have now inserted the unit for these (years).

*P18, L406 – A recent epidemiological study suggests…*

We have now corrected this spelling error.

*===========
More comments are included in the pdf text of the manuscript.*

We have gone through the pdf and corrected the additional comments within it.

*My opinion - the manuscript can be published after minor revision and corrections mentioned above.*

We again thank reviewer1 for a positive feedback and the many helpful comments.

*REVIEWER2*

*Reviewer #2: The Authors set up a factorial experiment to understand the effect of mesh size and drying condition in determining decomposition in litter bags along a gradient of naturally occurring radionuclides. The starting point are: the lack of knowledge about the effects of such naturally-occurring radionuclides, and the existence of a controversy in Chernobyl, where different studies have reported decreased litter decomposition in sites of high radioactive contamination, or a lack of such effects. The authors conducted a good amount of work in their experiment, as well as in estimating the transfer of different radionuclides, and their contribution to radiation dose to different soil organisms.*

We thank reviewer2 for acknowledging our work, and for commenting on our manuscript to improve it. We have tried to understand and respond to all the below comments, and we have tried to accommodate to all comments and suggestions that we interpret as constructive and that may improve the manuscript.

*However, the paper has fundamental shortcomings in all its major sections. The Introduction does not lay out a clear argument for the study, for example by explaining expected mechanisms for the reduction of decomposition following radiation exposure of the soil fauna. The Authors state in their abstract that they hypothesize naturally occurring radionuclides to affect soil fauna, and therefore decomposition. Yet they do not go into the precise mechanism –or alternative mechanisms – that might mediate such effect. They also routinely conflate radiation effects on abundance and diversity, and do not introduce the ecological roles of the different groups and their relevance to decomposition.*

The argument for the study in the introduction is that litterbags are a valid tool to assess the ecosystem-level parameter litter decomposition (by soil fauna), and effects of naturally occurring radionuclides on soil fauna thus will affect litter decomposition. We really believe that this is clear from the introduction. Our aim was not to investigate by which mechanisms natural radioactivity affects soil fauna, even though our results hint at toxicity (see our responses to other comments below on alternate explanations). To improve and further clear up our argument, we have included in the introduction a sentence about how litterbag decomposition is an ecosystem level parameter.

About us conflating radiation effects on abundance and diversity, this was mentioned in the discussion (L455), as also mentioned in the specific comments of reviewer2. Elsewhere we have mentioned diversity, it has been in relation to other stressors, citing the work/publications of others, and not comparing it directly to this work or effect on litterbag litter decomposition. One sentence where this could be misinterpreted was in introduction section 3, and this line has therefore now been moved to introduction section two, since it is about soil fauna response to chemicals. Regarding sentence (L455), we do agree that L455 in the originally submitted manuscript is somewhat speculative. It is also redundant, and we have therefore deleted it to accommodate to reviewer2.

Regarding the ecological roles of different groups and their relevance to decomposition, we have now gone through even more literature on soil fauna, litter decomposition and litterbags. As several authors report, soil fauna species often have overlapping ecological roles, as most soil fauna have a very generalist feeding behaviour and can adapt to abiotic and biotic changes (Briones 2014, Hedlund et al 2004, Menta 2012) and many authors rather use morphology traits regarding soil fauna being eu-edaphic (true soil dwellers) or epigeous (surface-dwellers, see fx da Silva et al 2016 in Applied soil Ecology, Ponge and Salmon 2013 in Paedobiologia). To clear up, we have included a sentence in the introduction about overlapping roles and adaptation ability of soil fauna.

*Surprisingly, the introduction does not even explain the specific results of previous studies (in Chernobyl and or elsewhere). Could variation among the results of previous studies be due to their methods? With the present paper being mostly methodological – which is fine –, I was surprised that the authors did not look more into the methods used by previous studies. The same is true for the effect of drying conditions, the other factor in their full factorial experiment.*

We believe that a discussion on different results between previous investigations belong in the discussion section, where these also can be related to differences or similarities with the results in the study at hand. To accommodate, we thus include an explanation about why the results of the two Chernobyl litterbag references differ in the discission section (see also comment by reviewer1).

In this part of the discussion, we focus on how the different results of these references probably are due to their different methodology with differences in level of radioactivity, spatial coverage, litter species, drying regime, litter bag mesh size, soil parameters and statistical analyses. We do not want to judge the results of other studies, so we only point out how some of these differences may have large effects on results. For example, different levels and ranges of radioactivity could be expected to affect their results, the very large mesh size of the litterbags in reference 34 allows also macrofauna into their litterbags, and the study at hand demonstrates that the different litter drying temperatures may affect decomposition.

Finally, with respect to anticipated effects of litter drying regime, we have now included more information about this (see also our response on the editors comment on denaturization).

*In a similar fashion, the discussion also does not leverage estimates about radiation exposure toward shedding light on the effects of radiation on litter decomposition, in spite of this being the reported objective of the study.*

To accommodate and better let the discussion leverage estimates of exposure to shed light on litter decomposition effects, we have now made some changes to our discussion. We now mention the levels of anthropogenic radioactivity in the two Chernobyl litterbag studies (L: ) for a comparison with our dose rates (see also our response an editor) with regard to levels and effects. With regard to leverage within our own study, expressed as variation between soil samples taken beneath litterbags and among the localities, we emphasize that this is both detailed in the text and shown for activity concentrations and dose rates among localities in figures 7, 8 and 12, as well as table 2.

We also emphasise that within the study area the magnitude of ^232^Th radionuclides is from 10 to 140 times higher than for ^238^U radionuclides. To make this absolutely clear, we have added a sentence to the results section (L: ). Also, to illustrate the differences in dose rates between these two decay series, we have now included subtotals for the radionuclides of the ^232^Th and ^238^U decay series, respectively, in table 3. We therefor found it very strange that in statistical models, variation in the activity concentration of ^226^Ra (ie the ^238^U series) explained variation in litter decomposition while variation in ^228^Ra (^232^Th series) did not. This combined with the very low effect size of dose rate (in the statistical models), around 1/3 of the effect size of activity concentration of ^226^Ra suggests that toxicity (probably ^238^U) is important to litter decomposition within the study area.

We did however think of yet another possible explanation that could leverage radiation exposure, that the concentration ratios (CR) applied in the ERICA tool (see methods section) were too low. Since the dose rate depends on both uptake (CR) but also half-life, higher CR values have a much larger impact on ^226^Ra than ^228^Ra dose rates (see table 3). If CR values really are much higher than assumed in the ERICA estimates, it could explain higher explanatory power of ^226^Ra (and the ^238^U series radionuclides), with regard to the different activity concentrations. To give our best efforts in accommodating to this comment by reviewer2, we have therefore also included a couple of sentences about this potential explanation to our results in the discussion.

*The analytical approach is also problematic. For example, I'm struggling to understand what it means to be comparing different statistical models that include estimates of radiation dose to different organisms as predictors. In other general, the authors are using model comparison wrong. The comparisons should be based on a priori alternative hypotheses about how to explain variation in decomposition among litter bags. For example, by comparing a model that only includes physicochemical variables (i.e. organic matter, pH,…), With one that includes these variables plus variables that describe the biological community, and or the radiation does that the different components of that community received.*

We do not fully understand this comment. In our experience, this is a common way to address a hypothesis regarding how to reveal what affects variation. We interpret that reviewer2 here by “a priori alternative hypotheses” refer to alternative statistical models. As we understand it, reviewer2 would have us compare our statistical models (of litter decomposition in litterbags we assume) with alternative models that includes either just “physiochemical variables” (what we term soil parameters) and models including also “variables that describe the biology community” and not radioactivity. We believe it is relatively clear from our methods section that we have not sampled any variables describing the biological community. That would involve a huge amount of work and we therefore did this investigation as a first and initial investigation of an ecosystem-level parameter, litter decomposition. Therefore, the only thing we can do to accommodate is to include a “null” model with only the soil parameters, something we have now done (table 5), which shows that such a null model is far “worse” than our statistical models. We also included a sentence in the methods and in the results chapters.

*Instead, the Authors did stepwise model simplification too. This stepwise removal of statistically nonsignificant factors is known to lead to statistical errors. This is because statistical tests assume a single step, and are inappropriate when sequential steps are taken. This is not a found statistical approach, irrespective of whether the Authors also look at AIC. (See Whittingham et al. 2006 Methods in Ecology and Evolution)*

Stepwise model simplification is to our knowledge a common approach. We have now read Whittingham et al 2006 in Animal Ecology, as well as several other publications on the topic. To accommodate to this concern of reviewer2 we have now included a new table (table 4) with the results of the full model versions of the two best models. This table shows the readers that the same terms (parameters) are significant in the full versions as are included in the simplified versions of these models. To avoid repeating results, one line is now removed and rephrased in the results. Moreover, it has been shown that parameter estimates are more accurate after stepwise regression (Hegyie and Laczi 2015) and we therefore maintain table 6 and have included a citation for this reference in the methods section.

*Re: the structure of the models, I have some other doubts. For example, it looks like the authors are analyzing the two different types of litter bags (as defined by their mesh size) separately (L 333 and following). I thought that one of the main points was to compare the two types of litter bags, and was expecting this to be a factor in these linear models. I also think that the linear models should include site and/or location within sight as random factors.*

We only sampled soil cores from beneath coarse meshed litterbags due to the cost and duration of HPGe gamma spectrometry. Therefore, we have only looked at covariation in soil parameters with litter decomposition in coarse litterbags (see also our response to a couple of the other comments by reviewer2). For the same reason, before these analyses, we did some simple comparisons to verify the soil fauna effect on litter decomposition (by comparing fine and coarse meshed bags). Thus we were also able to see the effect of oven-dried litter on microbes in the fine meshed bags, a point that supports our conclusions on a slower decomposition of oven-dried litter (see also our response to comments by editor). These results are also presented as box plots with clear differences. We hope this response satisfied reviewer2 and the editor.

*This brings me to another crucial problem with the analyses described by the authors. From their description of how they compared decomposition rate in the different litter bags, it looks like they compare each litter bag with each other one, therefore greatly inflating the number of their comparisons.*

We would like to emphasize two aspects that are crucial to this comment by reviewer2 (see also our response to a comment by the editor on sample size constraint). First, the very high degree of spatial heterogeneity among the naturally occurring radionuclides within the study area involves very large differences between even litterbags within the same locality (fx between quartets, see table 2). Also, we did not pool any soil samples, as the 50 soil cores taken beneath the 50 coarse meshed litterbags involved much variation in both radioactivity and soil parameters (see results and figures), which can be used to assess the variation in litter decomposition (among litterbags in statistical models). In our view this is relatively straight forward.

To accommodate and to clear up we have included an additional sentence about how this high spatial heterogeneity allows us to compare the variation in soil parameters (including radionuclides) among 50 coarse meshed litterbags with their variation in litter decomposition.

*Irrespective of the particular analyses chosen, the Authors do not clearly disentangle their results, or leverage them to advance understanding of the effect of . For example discriminating what explain the relevance of their results, and their*

We do not agree to this comment. Rather we believe that we through our analyses show that covariation of some soil parameters are very important and that while accounting for these parameters we show that parameters on radionuclides are significant, either in models on activity concentration or in dose rate models. Through these models we disentangle the importance of the uranium decay series compared to the thorium decay series (which is present at much higher levels), and we think we really have disentangled something. However, we hope that with our responses to the previous comments we have improved the view of reviewer2 on our manuscript and how we disentangle our results. We believe we now even better clarify the different effect from the two decay chains to improve our suggestion of toxicity effects.

*In addition, there is a certain lack of care throughout the paper, with several sentences that are broken (e.g., L 176) or riddled with typos*

We cannot understand what reviewer to refer to as being an error in L176. Through his comments, reviewer2 only points out two other such errors. Reviewer1 points out 7 typos but deems the text well written (see first comment by reviewer1). We apologise for the typos, but we do not agree that this qualifies for the term “being riddled with typos”.

*Some more specific comments follow.

Results

L 307 and following: All this variation in dosimetry, both within and among the different modeled organisms, is not analyzed or explained (it is also a missing component of the Discussion). It remains unclear whether such variation is due to uncertainty in the parameters, variation among different bags, or what.*

This is our chapter on results from the ERICA tool on the dosimetry of the four modelled organisms. Reviewer2 is wrong that we did not analyse or explain these results. These results are included in the explored statistical models. The summary statistics (ranges, means, median and SD) are presented in this chapter, which is the intention of this chapter. Moreover, in table 3 we summarize these results per radionuclide. The variation in each separate radionuclide (each cell) in table 3 is due to variation in activity concentration among soil samples (beneath coarse soil bags). We believe this is relatively clear from the methods section. The variation between radionuclides in table 3 (ERICA results) are within each model organism due to the properties of each radionuclide such as half-life and CR. The variation between model organisms are due to differences in organism assumptions (such as mass, chi and ksi) and differently assumed CR’s. We believe the background for these variances (commented on by reviewer2 and explained here) are relatively clear from the corresponding section in the methods section. It the reader should want to read up on the ERICA tool, we hav now provided two more references (Brown et al 2008 and Ulanovsky et al 2008, both in Journal of Environmental Radioactivity).

*Discussion

L 411: I suggested the authors actually lead with results in this paragraph. It's quite hard to follow the initial several sentences without a stated clear relationship with any result (which only appears on line 421). This paragraph will really benefit from a restructuring. Right now, it mentions several previous results, yet it never quite clarifies what the results of this paper add.*

We apologise, this chapter follows from the previous one (with results). To accommodate we have therefore combined this chapter with the previous (so lead is the results in question).

*This is also true – as for the Introduction – with regards to the relationship with previous results. The Authors mention again the existence of a controversy in Chernobyl, yet the present results are not put into that context, nor the Authors discuss how their results help advance the status of knowledge there.*

We now in the discussion compare the two Chernobyl litterbags studies, and their results to our results (see our responses to previous comments to avoid repetition). This should help the reader put our results into context.

*L 439: That the results indicate “an explanatory power of dose rate” is a vague way to put it. This is a common theme throughout. Results are often presented in this generic way, rather than explicitly indicating what the direction of the relationship is. (see for example: L 451: the study “identified effects”*

We guess reviewer two is referring to L441. We have searched through the document and the only place in the originally submitted manuscript we use the term “explanatory power” was in this line. We agree that is it always good to indicate the direction of a relationship and have now included that in these two sentences to accommodate. We do however like the term, as it reflects significance of parameter, so we have kept the term.

*L 445: ‘well below up to four times as high’ is a very confusing sentence.*

To accommodate we have rephrased.

*L 447: ‘Radioceasium’ should be ‘radiocaesium’*

We have now corrected this typo.

*L 449: I don’t understand this sentence. It is dose rates that are affecting soil fauna as opposed to what? Is this something about the dose rate as opposed to total overall dose?*

To clarify we have rephrased.

*L 455: I understand that the Authors did not measure biodiversity directly. Yet the relationship between biodiversity and decomposition is crucial to interpreting their results, as the Authors themselves hypothesize in the abstract and introduction, and acknowledge earlier in the discussion (e.g., L 443, when they talk about toxicity effects). So this should be expanded upon in the discussion. Right now, there is very little clarity as to what the results on litter decomposition indicate, as the Authors (1) do not sufficiently link them to the toxicity results (i.e., which taxa got the highest dose, and what that means for decomposition; which taxa were excluded by the mesh size, and what that means for decomposition; etc.).*

We agree that L455 in the originally submitted manuscript is somewhat speculative. It is also redundant, and we have therefore deleted it to accommodate to reviewer2. See also our answers and responses to previous comments by reviewer2 that cover the rest of this comment.

*Minor points

L 50: Agriculture is not an ecosystem function*

To accommodate we have now deleted agriculture.

*L 80: I never heard anyone referring to Chernobyl as "a Russian spill site”*

We apologise for the error. The cited author refers to spill sites across the old Soviet. We have now replaced “Russian” according to this comment by both reviewer1 and reviewer2.

*L 89: “does” should be “dose”*

This sentence reads “…Chernobyl litterbag results showing reduced decomposition [33] does not seem surprising,..” and refers to that these results “does not seem surprising”. This is in other words not a typo and should not be “dose”.

*L 90: what kind of effects? This seems fairly important given the expected role of soil fauna in mediating litter decomposition. Yet, this is only vaguely referred to here.*

We have now rephrased to accommodate.

*L 170: Xxx?*

We apologise, inserting the brand and model of the pH meter was one of the last things we did with the manuscript, and in doing so, we forgot to delete the text that was there initially to remind us of this. This was thus fill text meant to be replaced by the brand and model type. It has now been deleted.

*L 179: define secular equilibrium*

In our opinion secular equilibrium is a very well-established term and there should be no need to define it. To assess what has been the tradition in Plos ONE, we did a google search on “secular equilibrium” and “Plos ONE” and went through the pdfs of the first three hits (publications in Plos ONE). All these three publications use the term secular equilibrium without defining it. We therefore conclude that there is no need for a definition.

*L 199: Ksi and chi are not defined anywhere*

In ERICA an ellipsoid shape is assumed with a given ratio between width and length (Ksi) and a given ratio between height and width (Chi). To accommodate we have now included this information.

*L 213—226: this entire paragraph would better be reported in a table*

In our opinion there is all ready a high number of tables and figures, and we do not want to make a new one. We therefore suggest we keep this paragraph as it is.

*JOURNAL REQUIREMENTS*

*Journal Requirements:*

*When submitting your revision, we need you to address these additional requirements.*

*1. Please ensure that your manuscript meets PLOS ONE's style requirements, including those for file naming. The PLOS ONE style templates can be found at*

[*https://journals.plos.org/plosone/s/file?id=wjVg/PLOSOne_formatting_sample_main_body.pdf*](https://journals.plos.org/plosone/s/file?id=wjVg/PLOSOne_formatting_sample_main_body.pdf) *and*

[*https://journals.plos.org/plosone/s/file?id=ba62/PLOSOne_formatting_sample_title_authors_affiliations.pdf*](https://journals.plos.org/plosone/s/file?id=ba62/PLOSOne_formatting_sample_title_authors_affiliations.pdf)

We have now ensured that our revision meets PLOS ONE requirements according to the two above pdf’s. Figures will be uploaded as separate tiff files with 300 dpi and according to size limitations.

*2. In your Methods section, please provide additional information regarding the permits you obtained to collect samples for the present study. Please ensure you have included the full name of the authority that approved the field site access and, if no permits were required, a brief statement explaining why.*

This study was conducted in Norway, which do not have any system for field permits. Rather Norway has an act relating to outdoor recreation (friluftloven) under which “the right of public access” allows gathering of small amounts or samples. Moreover, since the study area is an area of high natural background radiation, field assessments falls under the jurisdiction of the Norwegian Radiation and Nuclear Safety Authority, for which this study was done. Local authorities and the landowner were accordingly notified. We have provided a short statement of this in the methods section. Moreover, the present study had negligible effects on the ecosystem functioning as the senescent fresh litter collected was only sampled at a very limited scale (which we also have included in the methods section).

*3. Thank you for stating the following in the Acknowledgments Section of your manuscript:*

*"This work was supported by the Research Council of Norway through its Centres of Excellence
465 funding scheme, project number 223268/F50. This was received thorough the Centre for
466 Environmental Radioactivity, CERAD, Centre of Excellence CoE."*

*We note that you have provided funding information that is not currently declared in your Funding Statement. However, funding information should not appear in the Acknowledgments section or other areas of your manuscript. We will only publish funding information present in the Funding Statement section of the online submission form.*

*Please remove any funding-related text from the manuscript and let us know how you would like to update your Funding Statement. Currently, your Funding Statement reads as follows:*

The funding statement should read as it did in the original acknowledgement section (below).

"This work was supported by the Research Council of Norway through its Centres of Excellence
funding scheme, project number 223268/F50. This was received by HH and RG at Norwegian Radiation and Nuclear Safety Authority (DSA) thorough the Centre for
Environmental Radioactivity, CERAD, Centre of Excellence CoE (https://www.nmbu.no/en/services/centers/cerad)."

*4. We note that you have stated that you will provide repository information for your data at acceptance. Should your manuscript be accepted for publication, we will hold it until you provide the relevant accession numbers or DOIs necessary to access your data. If you wish to make changes to your Data Availability statement, please describe these changes in your cover letter and we will update your Data Availability statement to reflect the information you provide.*

Yes, we have as stated now loaded our raw data to the Mendely data repository (DOI: 10.17632/384dttzrt3.1). Just now the dataset is under moderation at Mendely, but they will be opened to the public (same DOI) in a short while. After acceptance we will also include our R script to this repository (we await to be sure there are no more changes to the script due to more revisions).

*5. We note that Figure 1 in your submission contains map images which may be copyrighted. All PLOS content is published under the Creative Commons Attribution License (CC BY 4.0), which means that the manuscript, images, and Supporting Information files will be freely available online, and any third party is permitted to access, download, copy, distribute, and use these materials in any way, even commercially, with proper attribution. For these reasons, we cannot publish previously copyrighted maps or satellite images created using proprietary data, such as Google software (Google Maps, Street View, and Earth). For more information, see our copyright guidelines:* [*http://journals.plos.org/plosone/s/licenses-and-copyright*](http://journals.plos.org/plosone/s/licenses-and-copyright)*.*

*We require you to either (1) present written permission from the copyright holder to publish these figures specifically under the CC BY 4.0 license, or (2) remove the figures from your submission:*

1. *You may seek permission from the original copyright holder of Figure 1 to publish the content specifically under the CC BY 4.0 license.*

*We recommend that you contact the original copyright holder with the Content Permission Form (*[*http://journals.plos.org/plosone/s/file?id=7c09/content-permission-form.pdf*](about:blank)*) and the following text:*

*“I request permission for the open-access journal PLOS ONE to publish XXX under the Creative Commons Attribution License (CCAL) CC BY 4.0 (*[*http://creativecommons.org/licenses/by/4.0/*](about:blank)*). Please be aware that this license allows unrestricted use and distribution, even commercially, by third parties. Please reply and provide explicit written permission to publish XXX under a CC BY license and complete the attached form.”*

*Please upload the completed Content Permission Form or other proof of granted permissions as an "Other" file with your submission.*

*In the figure caption of the copyrighted figure, please include the following text: “Reprinted from [ref] under a CC BY license, with permission from [name of publisher], original copyright [original copyright year].”*

The map is NOT a reprint. It is a map generated in R using map data downloaded from Geonorge (see below), a webpage of the Norwegian Mapping Authority. These data originate from both Norwegian Mapping Authority and from Geological Survey of Norway. This has now been clarified in the methods section and the figure legend.

All map data used to generate the map in Figure 1 were downloaded from Geonorge (<https://kartkatalog.geonorge.no/metadata/8ecaa2d5-8b0a-46cf-a2a7-2584f78b12e2>), which is one of the web pages of the Norwegian Mapping Authority. The Norwegian Mapping Authority’s free products are licensed under [Creative Commons Attribution 4.0 International (CC BY 4.0)](http://creativecommons.org/licenses/by/4.0/). Their terms of use and instructions on copyright and use of material (<https://www.kartverket.no/en/api-and-data/terms-of-use>) states that: “Norwegian Mapping Authority’s free products, i.e. data, services and maps that have been released for free use for both commercial and non-commercial purposes. This means the name of the Norwegian Mapping Authority will appear in all contexts, where the products or extracts of these are used, be it applications, web solutions, printed products, illustrations or others in the following way: ©Kartverket. It should also be linked to our websites where this is possible.” We have therefore both 1) included the name of the Norwegian Mapping Authority in both the relevant methods section, in the acknowledgement section and in the legend of figure 1, and 2) included ©Kartverket onto figure 1 as required by their terms of use. We have also been in communication with the Norwegian Mapping Authority (Kartverket), getting in return a scanned and filled out content-permission-form with regard to copyright, which will be uploaded with our revision as “Other”. We hope that we have dealt with this matter in a satisfying manner. Our response is also copied into our cover letter as required in this comment.

The map data downloaded from Geonorge that orignates from Geological Survey of Norway (NGU) are under the Norwegian licence for public data: <https://data.norge.no/nlod/en/2.0/> We have been in communication also with NGU and got the content-permission-form filled out with regard to copyright, and we will upload also this as Other2. We have also cited and acknowledged NGU for the use of data in the methods and acknowledgements sections.

2. *If you are unable to obtain permission from the original copyright holder to publish these figures under the CC BY 4.0 license or if the copyright holder’s requirements are incompatible with the CC BY 4.0 license, please either i) remove the figure or ii) supply a replacement figure that complies with the CC BY 4.0 license. Please check copyright information on all replacement figures and update the figure caption with source information. If applicable, please specify in the figure caption text when a figure is similar but not identical to the original image and is therefore for illustrative purposes only.*

*The following resources for replacing copyrighted map figures may be helpful:*

*USGS National Map Viewer (public domain):* [*http://viewer.nationalmap.gov/viewer/*](about:blank)

*The Gateway to Astronaut Photography of Earth (public domain):* [*http://eol.jsc.nasa.gov/sseop/clickmap/*](about:blank)

*Maps at the CIA (public domain):* [*https://www.cia.gov/library/publications/the-world-factbook/index.html*](about:blank) *and* [*https://www.cia.gov/library/publications/cia-maps-publications/index.html*](about:blank)

*NASA Earth Observatory (public domain):* [*http://earthobservatory.nasa.gov/*](about:blank)

*Landsat:* [*http://landsat.visibleearth.nasa.gov/*](http://landsat.visibleearth.nasa.gov/)

*USGS EROS (Earth Resources Observatory and Science (EROS) Center) (public domain):* [*http://eros.usgs.gov/#*](about:blank)

*Natural Earth (public domain):* [*http://www.naturalearthdata.com/*](about:blank)

*Please include your amended statements within your cover letter; we will change the online submission form on your behalf.*
